# LRRC4 Deficiency Drives Premature Ovarian Insufficiency by Disrupting Metabolic Homeostasis in Granulosa Cells

**DOI:** 10.1002/advs.202417717

**Published:** 2025-05-02

**Authors:** Yujie Shang, Yunjun Li, Di Han, Kun Deng, Wei Gao, Minghua Wu

**Affiliations:** ^1^ The Affiliated Cancer Hospital of Xiangya School of Medicine Hunan Cancer Hospital Central South University Changsha 410000 China; ^2^ School of Chinese Medicine Hubei University of Chinese Medicine Wuhan 430065 China; ^3^ Hubei Shizhen Laboratory Wuhan 430060 China; ^4^ The Key Laboratory of Carcinogenesis of the Chinese Ministry of Health The Key Laboratory of Carcinogenesis and Cancer Invasion of the Chinese Ministry of Education Cancer Research Institute School of Basic Medicine Central South University Changsha 410078 China; ^5^ Affiliated Hospital of Hubei University of Chinese Medicine Wuhan 430061 China; ^6^ The First Affiliated Hospital of Henan University of Chinese Medicine Zhengzhou 450003 China; ^7^ Xiangya School of Public Health Central South University Changsha 410013 China

**Keywords:** granulosa cell, leucine rich repeat containing 4, metabolic homeostasis, mitochondria dynamics, premature ovarian insufficiency

## Abstract

Premature ovarian insufficiency (POI), defined by early loss of ovarian activity before the age of 40 years, is the leading cause of infertility and systematic aging in women, posing a public health challenge worldwide. However, its molecular etiology and therapeutic options are still lacking. Here, leucine‐rich repeat containing 4 (LRRC4) is identified as a critical regulator of folliculogenesis expressed in granulosa cells (GCs), which contributes to ovarian reserve maintenance. LRRC4 deficiency triggers defective oocyte maturation and excessive follicular atresia through inhibition of GC differentiation and ultimately leads to POI. Mechanistically, LRRC4 balances mitochondrial fission and fusion to inhibit excessive mitophagy by promoting the K48‐linked ubiquitination degradation of Yes‐associated protein (YAP), thereby maintaining the metabolic homeostasis of mitochondrial aerobic respiration and glycolysis. Importantly, targeting LRRC4 normalized follicular development and ovarian function in POI model mice. In conclusion, these data reveal the novel pathogenesis of POI and suggest that LRRC4 is a potential target for the diagnosis and treatment of POI.

## Graphical abstract

LRRC4 deficiency disrupts the metabolic homeostasis of mitochondrial aerobic respiration and glycolysis during GC differentiation by inhibiting the ubiquitination‐mediated degradation of YAP, which ultimately leads to POI. These findings reveal the novel molecular etiology of POI and provide a promising target for prevention and treatment.

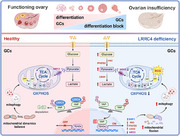

## 1 Introduction

Premature ovarian insufficiency (POI), previously termed premature ovarian failure (POF), refers to a clinical syndrome defined by loss of ovarian activity before the age of 40 years.^[^ ^1^ ^]^ Approximately 3%–5% of women are estimated to suffer from POI, and the incidence of this condition is progressively increasing, with a trend toward earlier onset.^[^ ^2^, ^3^, ^4^ ^]^ POI is not only the leading cause of female infertility, as the natural pregnancy rates of affected women are only 3%–10%,^[^ ^5^ ^]^ but also profoundly affects the skeletal, cardiovascular, and neurological systems, thereby accelerating systematic aging and increasing all‐cause mortality, which severely compromises women's health and well‐being.^[^ ^6^, ^7^ ^]^ However, to date, no therapeutic options are available to fundamentally protect or restore the ovarian reserve. Moreover, POI is often diagnosed too late, causing irreversible impairment in affected women.^[^ ^8^ ^]^ Given these limitations and issues, a better understanding of the pathogenesis of POI is crucial for early detection and effective interventions, which have important implications for promoting fertility and alleviating the effects of the aging of the global population.

Although the pathogenesis of POI is complex and highly heterogeneous,^[^ ^1^ ^]^ this process can be attributed primarily to abnormal follicular reserve and development, including a decreased primordial follicle pool, disturbed follicular recruitment/maturation, or accelerated follicular atresia. Folliculogenesis is a highly regulated process that depends on the mutual coordination of oocytes and cells surrounding them.^[^ ^9^ ^]^ Among these cell types, granulosa cells (GCs) are highly important, as they provide essential nutrients, growth factors, steroids, and the optimal microenvironment for oogenesis through an effective substance and signal exchange network.^[^ ^10^, ^11^, ^12^, ^13^ ^]^ Mitochondria in GCs play a pivotal role in follicular development and ovarian function through energy supply and redox balance maintenance.^[^ ^14^ ^]^ Mitochondrial dynamics serves as a critical mechanism for preserving mitochondrial homeostasis, which is essential for the functional networks that respond to metabolic demands.^[^ ^15^ ^]^ Dysregulated mitochondrial dynamics of GCs, particularly excessive mitochondrial fission, and insufficient fusion, is a key contributors to POI pathogenesis. Recent studies highlight that this imbalance impairs mitochondrial network integrity, disrupts energy metabolism, and exacerbates reactive oxygen species (ROS) overproduction. These pathological alterations further trigger GC apoptosis, thereby accelerating follicular atresia and ovarian reserve depletion, ultimately leading to POI and ovarian aging.^[^ ^16^, ^17^ ^]^

Emerging studies indicate that YAP signaling is pivotal in governing follicular development.^[^ ^18^ ^]^ In GCs, the active form of YAP is predominantly expressed in the nucleus to trigger proliferation, whereas the inactive form of YAP is mainly localized to the cytoplasm to modulate differentiation.^[^ ^19^, ^20^ ^]^ Aberrant accumulation of nuclear YAP not only triggers excessive activation of primordial follicles but also induces GC dedifferentiation and reprogramming, accelerating follicular atresia and ovarian reserve depletion,^[^ ^21^ ^]^ ultimately leading to POI.^[^ ^22^ ^]^ Furthermore, previous studies have demonstrated that YAP plays a critical role in regulating the transcription of key regulatory factors regarding mitochondrial dynamics and energy metabolic processes. Dysregulated YAP activity disrupts mitochondrial homeostasis, resulting in an impaired metabolic pattern characterized by defective oxidative phosphorylation (OXPHOS).^[^ ^23^, ^24^, ^25^, ^26^ ^]^ Although YAP dysregulation in GCs has been implicated in the pathogenesis of POI, the mechanisms underlying its dysregulation, as well as the role of YAP in maintaining the homeostasis of mitochondria and metabolism within the follicular microenvironment remain largely elusive.

Leucine‐rich repeat containing 4 (LRRC4), also known as Netrin‐G ligand‐2 (NGL‐2), is a transmembrane and cytoplasmic protein that plays a critical role in cell proliferation, differentiation, and development through complex and precise cascades of signaling events.^[^ ^27^, ^28^, ^29^, ^30^ ^]^ LRRC4 is expressed in human and mouse ovaries.^[^ ^31^ ^]^ Here, we identified LRRC4 as a key regulatory factor of folliculogenesis that is expressed in GCs and contributes to the ovarian reserve. By employing GCs from LRRC4 knockout mice and KGN cells (a human granulosa‐like tumor cell line), as well as a cyclophosphamide (CTX)‐induced POI model, our study reveals the novel pathogenesis of POI that LRRC4 balances mitochondrial fission and fusion to maintain mitochondrial metabolic homeostasis by promoting the ubiquitination degradation of YAP. These findings not only advance the understanding of pathogenesis but also highlight LRRC4 as a promising target for the prevention and treatment of POI.

## 2 Results

### 2.1 LRRC4 is Involved in Folliculogenesis

**Figure 1:**
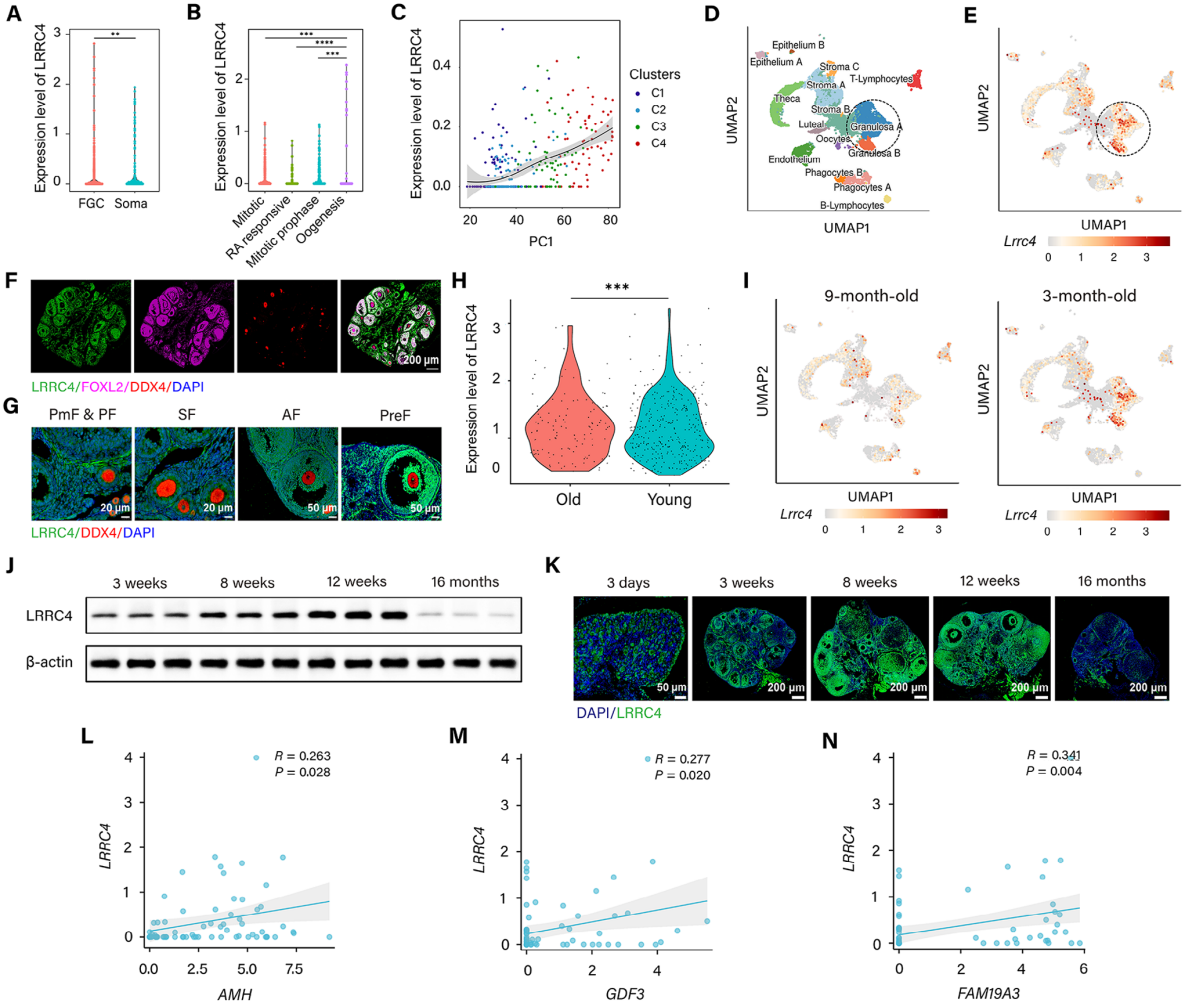
The dynamic expression pattern of LRRC4 in the ovary. A and B) Expression of LRRC4 in fetal germ cells (FGCs) and gonadal somatic cells (A) and different development phases of FGCs (B) based on GSE86146. Student's t‐test and one‐way ANOVA. C) Expression of LRRC4 in human ovarian cells during folliculogenesis based on GSE107746. C1, Primary Follicle; C2, Secondary Follicle; C3, Antral Follicle; C4, Preovulatory Follicle. D and E) Feature plots showing subsets in mouse ovarian cells (D) and LRRC4 expression in these subsets (E) based on GSE86146. F) Representative fluorescent image showing co‐expression of LRRC4 (green), FOXL2 (violet), and DDX4 (red) in mouse ovaries. Scale bar, 200 µm. G) Representative fluorescent images of LRRC4 expression in mouse ovaries during folliculogenesis. PmF, Primordial Follicle; PF, Primary Follicle; SF, Secondary Follicle; AF, Antral Follicle; PreF, Preovulatory Follicle. Scale bar for PmF, PF, and SF, 20 µm; Scale bar for AF and PreF, 50 µm. H) LRRC4 expression in ovarian cells from young and perimenopausal women based on GSE202601. Student's *t*‐test. I) LRRC4 expression in ovarian cells from mice at 3‐month‐old and 9‐month‐old based on GSE232309. J) LRRC4 protein expression of mouse ovaries from different ages (*n* = 3 biological replicates). K) Representative fluorescent images of LRRC4 expression in mouse ovaries from different ages. Scale bar for 3 days, 50 µm; Scale bar for 3, 8, 12 weeks and 16 months, 200 µm. L and N) Correlation analysis of LRRC4 expression with biomarkers of ovarian reserve. Data are presented as mean ± SEM. *p < 0.05, **p < 0.01, ***p < 0.001, ****p < 0.0001.

We initially investigated the expression pattern of LRRC4 in ovaries. On the basis of published single‐cell and transcriptomic sequencing data from human embryos and ovaries (GSE86146, GSE107746), we found that LRRC4 maintains its expression throughout follicular development. During embryonic development, LRRC4 is expressed in both fetal germ cells (FGCs) and gonadal somatic cells (**Figure** **1**A). Intriguingly, as development progressed, LRRC4 levels in female FGCs gradually increased from the early (mitotic phase) to late phases (RA signaling responsive, meiotic prophase, oogenesis phase) (Figure 1B). Similar to the changes in embryos, the expression of LRRC4 increased progressively during folliculogenesis and peaked in antral follicles (Figure 1C). Data from mouse single‐cell sequencing revealed high expression of LRRC4 in GCs (Figure 1D,E), which was further confirmed in mouse ovaries, as LRRC4 was coexpressed with FOXL2 (a typical GC marker) (Figure 1F). Consistent with the dynamic expression pattern observed in human ovaries, LRRC4 was detected in mouse GCs at all stages of follicular development and progressively increased, peaking in antral follicles (Figure 1G).

Given that age is a critical indicator for evaluating follicular quality and ovarian function, we next investigated whether LRRC4 expression changes with age. Notably, young females presented higher levels of LRRC4 than advanced did (GSE202601, GSE232309) (Figure 1H,I), indicating that LRRC4 may play a role in ovarian aging. We further examined the expression pattern of LRRC4 in mouse ovarian tissues from different ages. An age‐associated decrease in LRRC4 expression was also detected in mice, as LRRC4 expression significantly increased from 3 days to 12 weeks after birth (equivalent to the age range from 2 months to 24 years in humans), but it markedly decreased at 16 months of age (equivalent to 50 years in humans) (Figure 1J,K). Furthermore, we analyzed the relationship between LRRC4 levels and the ovarian reserve and found that LRRC4 was positively correlated with biomarkers of the ovarian reserve (Figure 1L‒N). Taken together, our results preliminarily reveal the expression pattern of LRRC4, indicating that LRRC4 is a key regulatory factor of follicular development and positively regulates ovarian reserve.

### 2.2 LRRC4 Deficiency Leads to POI

**Figure 2:**
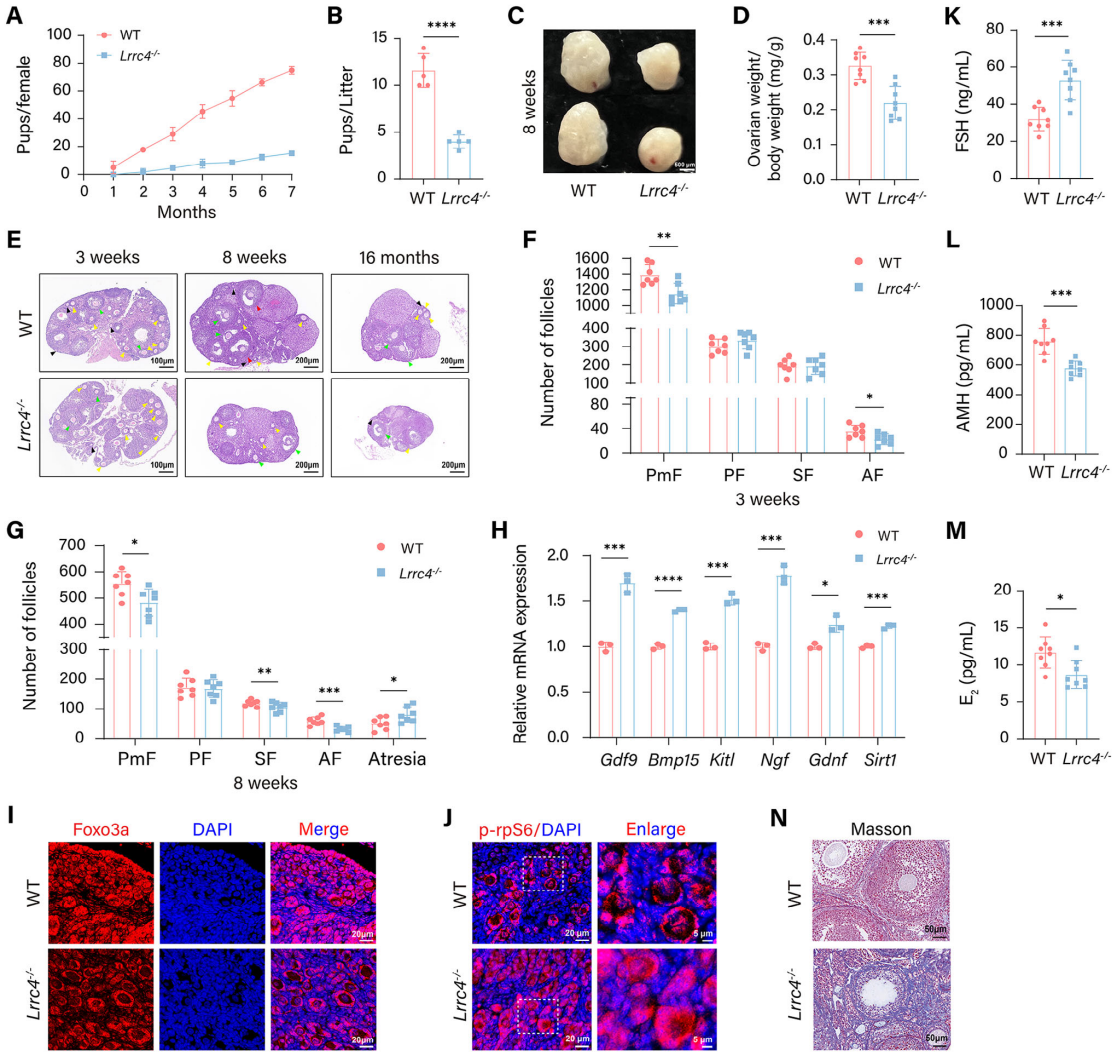
LRRC4 deficiency disturbs follicular development leading to premature ovarian insufficiency. A) Cumulative number of offspring in WT and *Lrrc4^−/−^* mice (*n* = 5 mice per group). B) Average litter size of WT and *Lrrc4^−/−^* mice (*n* = 5 mice per group). C) Representative images of ovarian morphology from 8‐week‐old WT and *Lrrc4^−/−^* mice. D) Ovary index values of 8‐week‐old WT and *Lrrc4^−/−^* mice (*n* = 8 mice per group). E) Representative images of ovaries from WT and *Lrrc4^−/−^* mice at 3‐week‐old, 8‐week‐old and 16‐month‐old by HE staining. Black arrows: PmF, yellow arrows: PF, green arrows: SF, and red arrows: AF. Scale bar for 3 weeks, 100 µm; Scale bar for 8 weeks and 16 months, 200 µm. F and G) Numbers of follicles in different developmental stages per ovary in 3‐week‐old (F) and 8‐week‐old WT and *Lrrc4^−/−^* mice (*n *= 7 mice per group). H) Relative mRNA expression of genes triggering primary follicle activation in ovaries from 3‐week‐old WT and *Lrrc4^−/−^* mice (*n* = 3 biological replicates). (I and J) Representative fluorescent images of Foxo3a I) and p‐rpS6 J) expression in 3‐week‐old WT and *Lrrc4^−/−^* mice. Scale bar, 20 µm. K to M) Serum levels of FSH (K), AMH (L) and E_2_ N) in 8‐week‐old WT and *Lrrc4^−/−^* mice (*n* = 8 mice per group). (N) Representative Masson staining images of ovaries from 8‐week‐old WT and *Lrrc4^−/−^* mice. Scale bar, 50 µm. Results are calculated with the Student's *t*‐test. Data are presented as mean ± SEM. *p < 0.05, **p < 0.01, ***p < 0.001, ****p < 0.0001.

We further clarified how LRRC4 affects folliculogenesis and ovarian function in LRRC4 knockout (*Lrrc4^−/−^*) mice. Progressive loss of fertility is considered the most intuitive result of ovarian insufficiency; hence, we first evaluated the fertility of wild‐type (WT) and *Lrrc4^−/−^* mice. Compared with the WT littermates, the *Lrrc4^−/−^* females had fewer pups and smaller litter sizes (**Figure** **2**A,B). Interestingly, the ovaries of both genotypes were indistinguishable in size at 3 weeks of age (Figure S1A,B, Supporting Information), while the ovaries of the 8‐week‐old *Lrrc4^−/−^* mice were obviously smaller and had a lower ovarian index than those of the WT controls (Figure 2C‒E). According to the results of follicular quantification, the number of primordial follicles in the 3‐week‐old *Lrrc4^−/−^* mice markedly decreased (Figure 2F). Similarly, a reduced number of primordial and growing follicles (including secondary and antral follicles) with more atretic follicles was observed in the 8‐week‐old and 16‐week‐old *Lrrc4^−/−^* mice (Figure 2G; Figure S2, Supporting Information), indicating that LRRC4 deficiency caused excessive follicular activation and atresia. Assessments of the key regulators of follicular activation further validated the phenotype noted above, as the *Lrrc4^−/−^* mice presented increased levels of regulators contributing to follicular activation accompanied by increased cytoplasmic FOXO3A and phosphorylated rpS6 levels (Figure 2H‒J). The elevated follicle‐stimulating hormone (FSH) and reduced anti‐Mullerian hormone and estradiol (E_2_) levels in the *Lrrc4^−/−^* mice also supported the ovarian insufficiency induced by LRRC4 deficiency (Figure 2K‒M). In addition, increased ovarian fibrosis was observed in the stroma of the *Lrrc4^−/−^* ovaries (Figure 2N). Overall, these results suggest that LRRC4 deficiency causes excessive follicular activation and atresia, which accelerates ovarian reserve loss and ultimately leads to POI.

### 2.3 LRRC4 Deletion Disrupts GC Differentiation

**Figure 3:**
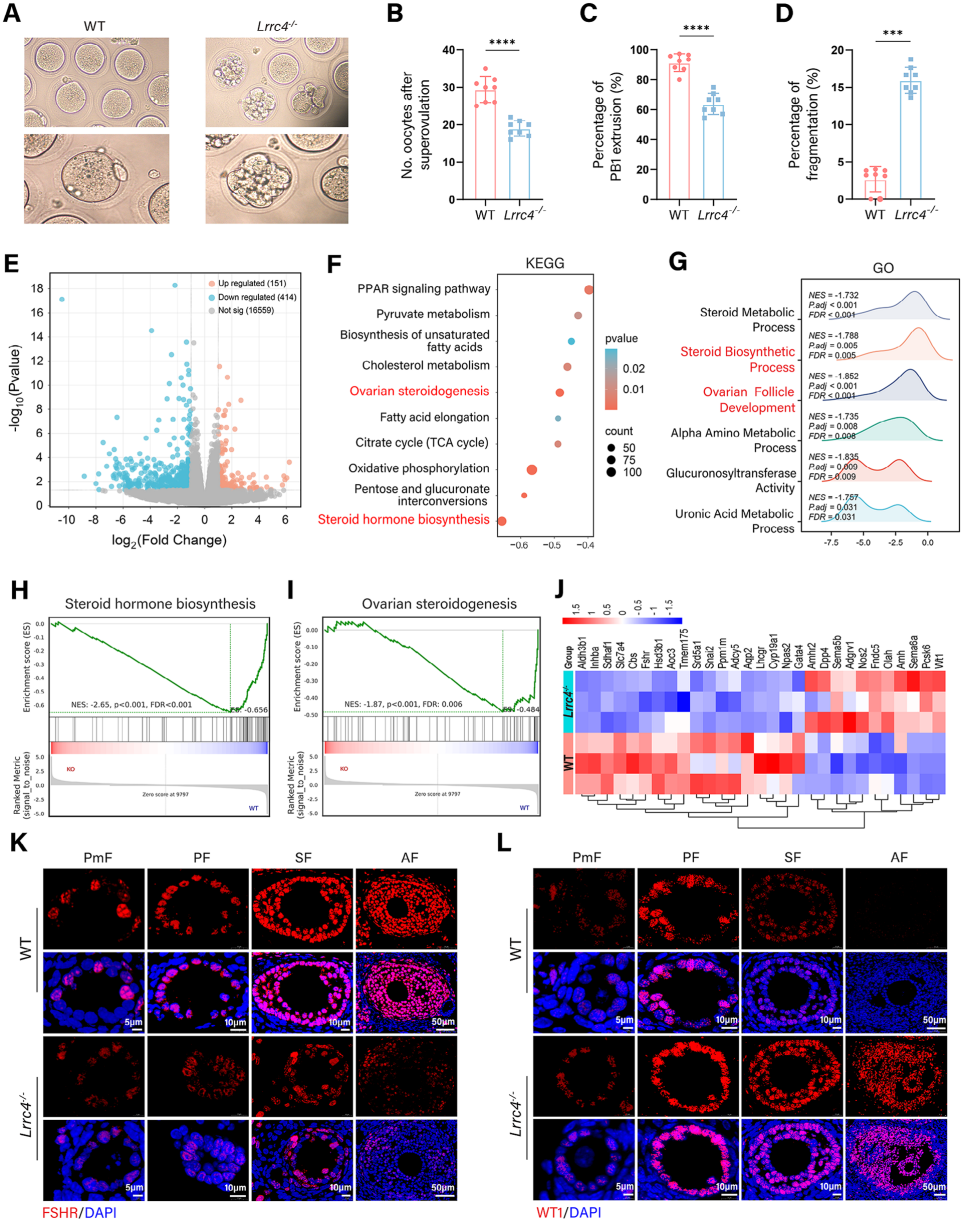
LRRC4 deficiency induces oocyte maturation defects by inhibiting granulosa cell differentiation. A) Representative images of in vivo matured oocytes collected from 3‐week‐old WT and *Lrrc4^−/−^* mice. Scale bar, 50 µm; magnified scale bar, 30 µm. B) Number of ovulated oocytes from 3‐week‐old WT and *Lrrc4^−/−^* mice (*n* = 8 mice per group). C) The percentage of PB1 extrusion from 3‐week‐old WT and *Lrrc4^−/−^* mice (n = 8 mice per group). D) The proportion of oocyte fragmentation from 3‐week‐old WT and *Lrrc4^−/−^* mice (*n* = 8 mice per group). E) The volcano plot displaying differentially expressed genes (DEGs) in GCs from 8‐week‐old WT and *Lrrc4^−/−^* mice (*n* = 3 mice per group). FC, fold change; FDR, false discovery rate. F and G) KEGG (F) and GO (G) analysis of DEGs in GCs from WT and *Lrrc4^−/−^* mice. H and I) GSEA enrichment analysis of the steroid hormone biosynthesis (H) and ovarian steroidogenesis (I) based on transcriptomics in GCs from WT and *Lrrc4^−/−^* mice. J) Heatmap showing biomarkers of GCs differentiation based on transcriptomics in GCs from WT and *Lrrc4^−/−^* mice. K and L) Representative fluorescent images of FSHR (K) and WT1 (L) in mouse ovaries from WT and *Lrrc4^−/−^* mice. Scale bar for PmF, 5 µm; Scale bar for PF and SF, 10 µm; Scale bar for AF, 50 µm. Results are calculated with the Student's *t*‐test. Data are presented as mean ± SEM. ***p < 0.001, ****p < 0.0001.

Folliculogenesis is a remarkably complex and well‐orchestrated process that relies on synchronization between oocyte maturation and the growth of neighboring GCs. Therefore, we investigated the effects of LRRC4 on oocytes and GCs. For this purpose, we evaluated the number and morphology of oocytes collected from 3‐week‐old mice after superovulation. As shown in **Figure** **3**B,C, the number of ovulated oocytes and the maturation rate determined by extrusion of the first polar body (PB1) dramatically decreased in the *Lrrc4^−/−^* mice. Oocytes from the *Lrrc4^−/−^* mice displayed abnormal morphology with a significantly greater incidence of fragmentation (Figure 3A,D). To evaluate the developmental potential of *Lrrc4^−/−^* oocytes, germinal vesicle (GV) oocytes isolated from 3‐week‐old *Lrrc4^−/−^* and the control mice were cultured in vitro. Interestingly, no significant difference was observed in the proportion of oocytes attaining the MII stage between groups (Figure S3, Supporting Information). These findings demonstrated that LRRC4 deficiency impairs oocyte maturation primarily by disrupting the follicular microenvironment, rather than from any intrinsic defects in the oocyte maturation potential.

GCs are crucial for oocyte maturation and follicular development, as they provide nutrients and mechanical support for oocytes. Given that LRRC4 is highly expressed in GCs, we focused on GCs to further investigate the role of LRRC4 in folliculogenesis. We found that the fluorescence signals of terminal deoxynucleotidyl transferase dUTP nick end labeling (TUNEL), caspase 3, and γ‐H2AX were prominently elevated in GCs from the *Lrrc4^−/−^* mice (Figure S4A‒C, Supporting Information). Comparatively, no evident differences in PCNA immunostaining were detected between the groups (Figure S4D, Supporting Information), suggesting that LRRC4 deficiency promotes apoptosis and DNA damage in GCs. To further explore the mechanisms underlying LRRC4‐dependent folliculogenesis, we performed RNA sequencing (RNA‐seq) analysis of GCs from *Lrrc4^−/−^* and WT mice. A total of 567 differentially expressed genes (DEGs) were identified (|log_2_FC|>1 and *P* < 0.05) between the *Lrrc4^−/−^* and WT groups, with 151 genes upregulated and 416 genes downregulated (Figure 3E). Gene Ontology (GO) and Kyoto Encyclopedia of Genes and Genomes (KEGG) enrichment analyses revealed that LRRC4 deficiency was associated primarily with steroid hormone biosynthesis, OXPHOS, ovarian steroidogenesis, the tricarboxylic acid (TCA) cycle, the PPAR signaling pathway, pyruvate metabolism, fatty acid biosynthesis, and cholesterol metabolism (Figure 3F,G). The leading‐edge plot of gene set enrichment analysis (GSEA) further revealed the inhibition of steroid hormone biosynthesis and ovarian steroidogenesis (Figure 3H,I). Mural granulosa cells derived from GCs, highly express gonadotropin receptors and are primarily responsible for the secretion of steroid hormones. Hence, we hypothesized that defective GC differentiation could be responsible for the impaired ovarian steroidogenesis induced by LRRC4 deficiency. Notably, the *Lrrc4^−/−^* group presented substantially inhibited expression of biomarkers of GC differentiation (Figure 3J). Immunofluorescence staining of mouse ovaries showed that the FSHR decreased progressively while WT1 increased gradually across follicular growth in the *Lrrc4^−/−^* group. Elevated WT1 inhibits the expression of steroidogenesis‐related genes, thereby further disrupting GC differentiation (Figure 3K,L).

Collectively, these results suggest that LRRC4 promotes GC differentiation to regulate oocyte maturation and contributes to folliculogenesis.

### 2.4 LRRC4 Knockout Drives Metabolic Reprogramming in GCs

**Figure 4:**
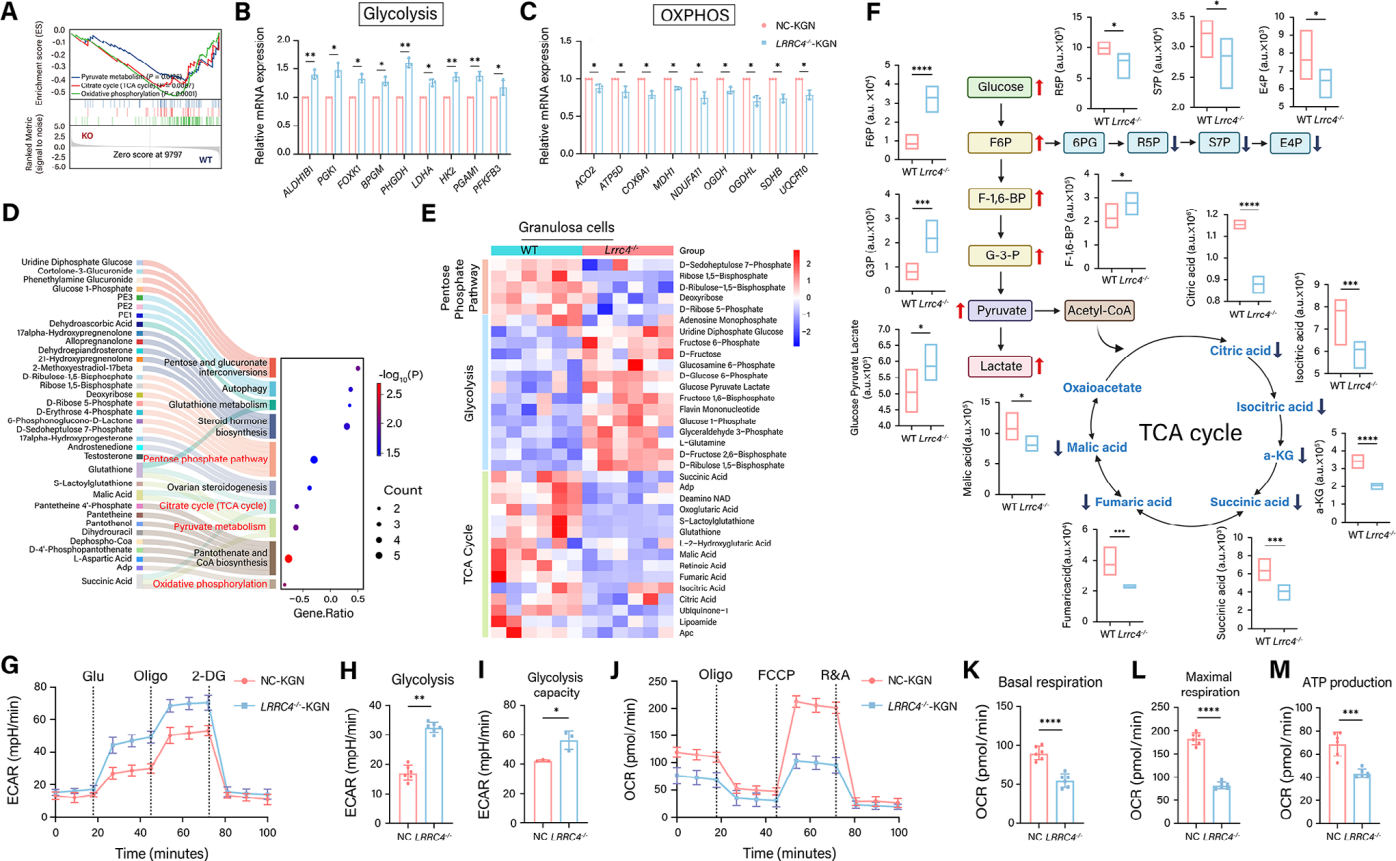
LRRC4 deficiency drives metabolic reprogramming in GCs. A) GSEA enrichment analysis of the pyruvate metabolism, TCA cycle, and oxidative phosphorylation (OXPHOS) based on the transcriptome of GCs from WT and *Lrrc4^−/−^* mice. B and C) Relative mRNA expression of glycolysis (B) and OXPHOS (C) genes (*n* = 3 biological replicates). D) Sankey diagram showing GSEA enrichment analysis based on metabolomics in GCs from WT and *Lrrc4^−/−^* mice. E) Heatmap of differential metabolites in glycolysis, TCA cycle and pentose phosphate pathway (PPP) in GCs from WT and *Lrrc4^−/−^* mice. F) Relative levels of the major metabolites in glycolysis and TCA cycle in GCs from WT and *Lrrc4^−/−^* mice. G to M) Real‐time seahorse ECAR and OCR measurements for glycolysis and OXPHOS speed evaluation in NC and *LRRC4^−/−^* KGN cells. Results are calculated with the Student's *t*‐test. Data are presented as mean ± SEM. *p < 0.05, **p < 0.01, ***p < 0.001, ****p < 0.0001.

The results of the GO and KEGG enrichment analyses revealed that LRRC4 is involved in GC metabolism (Figure 3F,G). GSEA further revealed the suppression of pyruvate metabolism, OXPHOS, and the TCA cycle (**Figure** **4**A). To elucidate the mechanism underlying LRRC4 deficiency‐induced metabolic disorders, we utilized KGN cells to establish an LRRC4 knockout model (*LRRC4^−/−^* KGN) for in vitro studies. Real‐time qPCR assays verified the substantial changes in glycolytic and mitochondrial OXPHOS genes between the groups, revealing a metabolic switch characterized by low OXPHOS and elevated glycolysis in *LRRC4^−/−^* KGN cells (Figure 4B,C). To confirm this phenotype, we conducted a metabolomic study to compare the metabolic profiles of GCs from WT and *Lrrc4^−/−^* mice. GSEA of the metabolomic data revealed that the altered metabolic profiles induced by LRRC4 loss were associated mainly with pentose and glucuronate interconversions, the pentose phosphate pathway (PPP), pyruvate metabolism, the TCA cycle, pantothenate and CoA biosynthesis, OXPHOS, ferroptosis and autophagy (Figure 4D). We further analyzed major metabolites of glycolysis and mitochondrial aerobic respiration and found dramatically elevated glycolytic activity in GCs lacking LRRC4, which was supported by increased levels of metabolites, including glucose, fructose 6‐phosphate (F6P), fructose 1,6‐bisphosphate (F‐1,6‐BP), glyceraldehyde 3‐phosphate (G3P), pyruvate and lactate (Figure 4E,F). Conversely, metabolites associated with the PPP and TCA cycle, such as ribose 5‐phosphate (R5P), erythrose 4‐phosphate (E4P), sedoheptulose 7‐phosphate (S7P), citrate, malate, α‐ketoglutarate (α‐KG), succinate and fumarate, were present at reduced levels (Figure 4E,F), indicating damaged OXPHOS. As a key metabolic link between glycolysis and the TCA cycle, pyruvate can be oxidized to produce adenosine triphosphate (ATP), NADH, and H_2_O during mitochondrial aerobic respiration or fermented to lactate under anaerobic conditions. The accumulation of pyruvate and lactate in the *Lrrc4^−/−^* group also demonstrated that LRRC4 knockout impaired mitochondrial OXPHOS. These findings were further validated in KGN cells. Changes in the extracellular acidification rate (ECAR) were associated with increased basal glycolysis and glycolytic capability in *LRRC4^−/−^* cells (Figure 4G‒I), indicating increased glucose utilization following LRRC4 ablation. A cell mitochondrial stress test in which the oxygen consumption rate (OCR) in *LRRC4^−/−^* KGN cells was monitored demonstrated significant reductions in basal respiration, ATP production, and maximal oxygen consumption, suggesting mitochondrial respiration deficits (Figure 4J‒M). Hence, LRRC4 could serve as a potent regulator of mitochondrial aerobic respiration. LRRC4 deficiency impaired mitochondrial aerobic respiration and induced redox imbalance, thus driving metabolic reprogramming from OXPHOS to compensatory glycolysis.

### 2.5 LRRC4 Ablation Triggers Imbalanced Mitochondrial Dynamics

**Figure 5:**
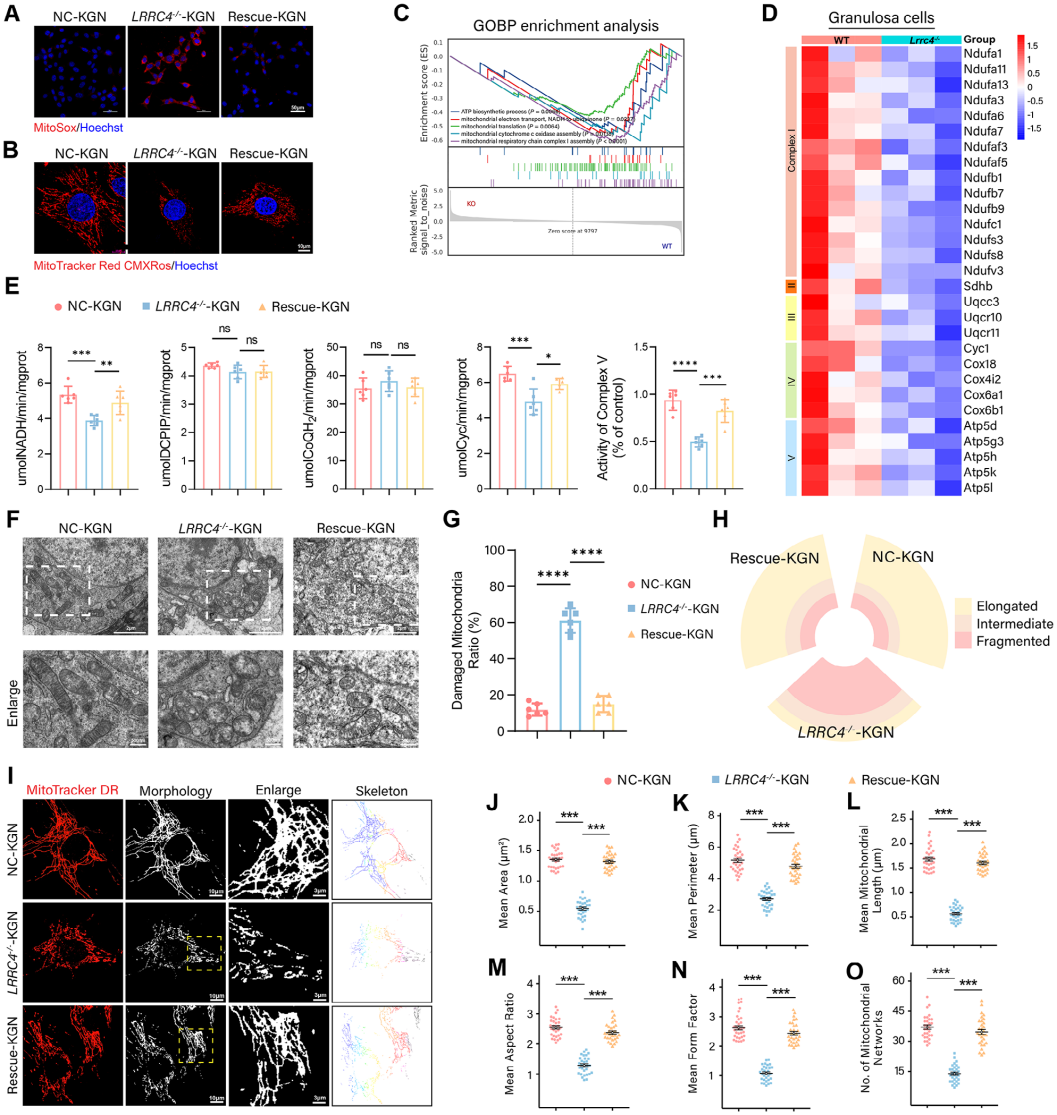
LRRC4 deficiency impairs mitochondrial structure and function. A) Representative fluorescent images of mtROS levels by MitoSox staining in NC, *LRRC4^−/−^* and Rescue KGN cells. Scale bar, 50 µm. Rescue KGN cells refer to the reintroduction of LRRC4 in *LRRC4^−/−^* KGN cells. B) Representative fluorescent images of mitochondrial membrane potential levels by MitoTracker Red CMXRos staining in NC, *LRRC4^−/−^* and Rescue KGN cells. Scale bar, 10 µm. C) GSEA enrichment analysis of the ATP biosynthetic process, mitochondrial electron transport (NADH to ubiquinone), mitochondrial translation, mitochondrial cytochrome c oxidase assembly and mitochondrial respiratory chain complex I assembly based on transcriptome of GCs from WT and *Lrrc4^−/−^* mice. D) Heatmap of DEGs in mitochondrial respiratory chain complex subunits based on transcriptomics in GCs from WT and *Lrrc4^−/−^* mice. E) Assessment on the activity of mitochondrial respiratory chain complexes in NC, *LRRC4^−/−^* and Rescue KGN cells (*n* = 6 biological replicates). F to H) Analysis of mitochondria structure and morphology in NC, *LRRC4^−/−^* and Rescue KGN cells by transmission electron microscopy. Representative electron microscopy images of mitochondria (F), damaged mitochondria ratio (G), and different mitochondria morphology ratios (H) per microscopic field (*n* = 6 biological replicates). Scale bar, 2 µm; magnified scale bar, 500 nm. I to O) Analysis of mitochondria morphology in NC, *LRRC4^−/−^* and Rescue KGN cells by confocal microscopy. Representative fluorescent images of mitochondria morphology (I) and relevant parameters, including mitochondrial average area (J), perimeter (K), length (L), aspect ratio (M), form factor (N) and number of mitochondrial networks (O) (n = 30 biological replicates). Scale bar, 10 µm; magnified scale bar, 3 µm. Results are calculated with one‐way ANOVA. Data are presented as mean ± SEM. *p < 0.05, **p < 0.01, ***p < 0.001, ****p < 0.0001, ns, not significant.

The dramatic suppression of mitochondrial OXPHOS caused by LRRC4 deletion prompted us to explore the role of LRRC4 in modulating mitochondrial structure and function. We first evaluated the status of mitochondrial reactive oxygen species (mtROS) and the mitochondrial membrane potential (MMP) to assess mitochondrial mass and OXPHOS efficiency and found that LRRC4 deletion significantly increased mtROS and decreased the MMP. LRRC4 reintroduction substantially reduced mtROS levels and partially rescued the MMP (**Figure** **5**A,B). The mitochondrial respiratory chain is critical for maintaining redox balance and MMP stability. GSEA of the RNA‐seq data suggested that LRRC4 deficiency‐induced mitochondrial dysfunction was associated with mitochondrial respiratory chain assembly, mitochondrial electron transport, and mitochondrial translation (Figure 5C). Given this, we next examined the function of the mitochondrial respiratory chain. The majority of DEGs associated with mitochondrial respiratory chain complex subunits were strongly suppressed following LRRC4 knockout (Figure 5D). *LRRC4^−/−^* cells presented a significant decrease in the activity of complexes I, IV, and V, which could be restored by reintroducing LRRC4 (Figure 5E). The mitochondrial crista, the highly folded inner mitochondrial membrane, is the location where the mitochondrial respiratory chain exists. The shape and structure of mitochondria determine the distribution and capacity of the mitochondrial respiratory chain. For this purpose, transmission electron microscopy (TEM) was used to observe the differences in mitochondrial ultrastructure between the groups. In the WT group, the mitochondria were all regular tubules with prominent cristae and intact membranes, whereas the mitochondria from *LRRC4^−/−^* cells were swollen and fragmented with broken or underdeveloped cristae, typical structural changes of increased mitochondrial fission and attenuated fusion. The reintroduction of *LRRC4* restored crista formation and normalized mitochondrial morphology (Figure 5F‒H). We then visualized the mitochondria in KGN cells by confocal microscopy. Networks of elongated tubule mitochondria, which were predominantly localized in the perinuclear region, were observed in the control group, whereas *LRRC4^−/−^* cells presented few mitochondrial networks with circular and small mitochondria dispersed in the cytoplasm (Figure 5I). Compared with the control, LRRC4 ablation significantly reduced the average mitochondrial area, perimeter, length (Figure 5J‒L), and number of mitochondrial networks (Figure 5O) but increased mitochondrial circularity, as revealed by a marked decrease in the aspect ratio and form factor (Figure 5M,N). The reintroduction of LRRC4 effectively normalized the morphology and distribution of mitochondria. These results demonstrated that LRRC4 deficiency disrupted mitochondrial morphology and structure, leading to impairment of the mitochondrial respiratory chain and OXPHOS, which in turn exacerbated mtROS accumulation and MMP collapse.

**Figure 6:**
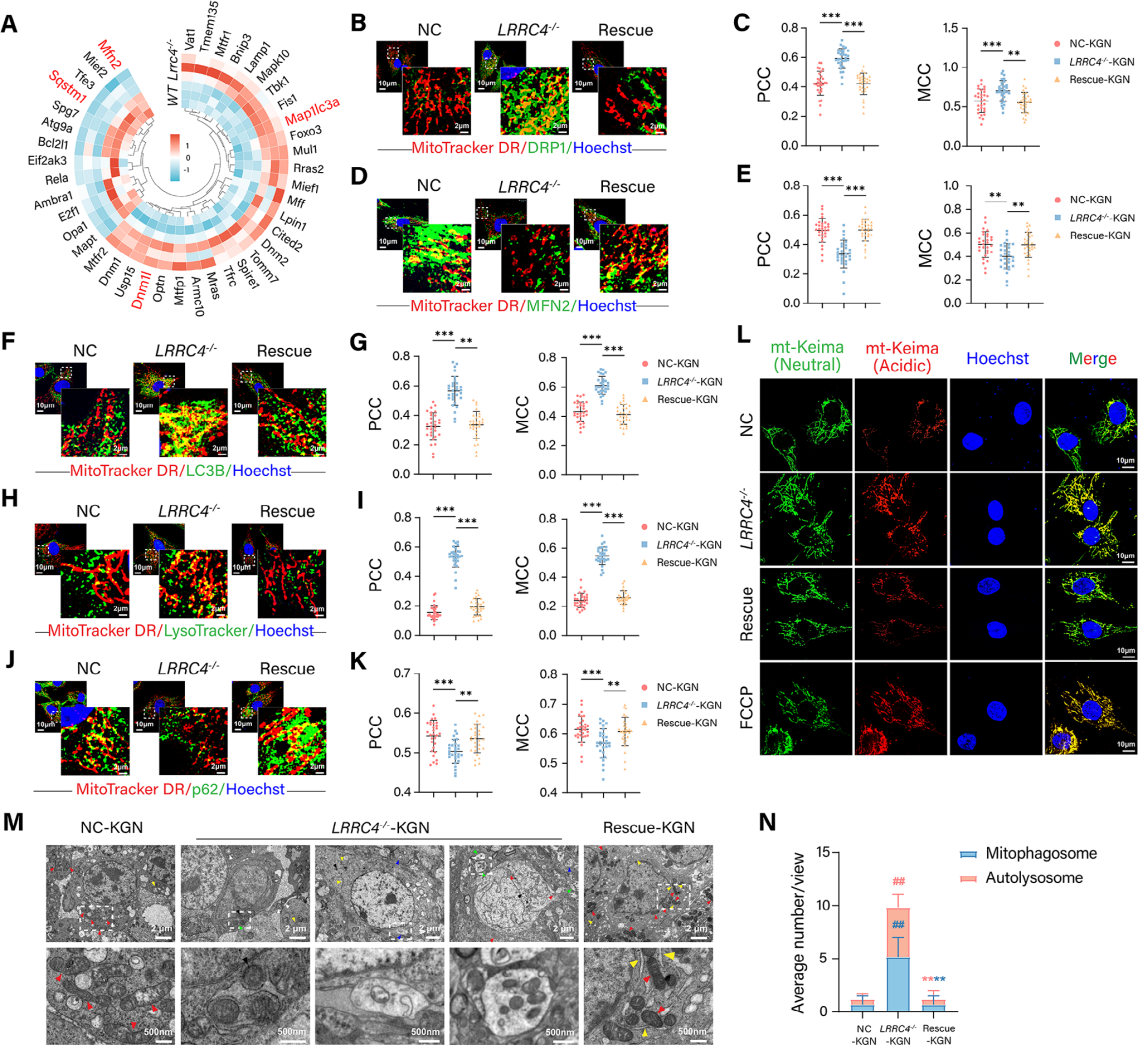
LRRC4 deficiency triggers imbalance in mitochondrial dynamics. A) Heatmap of DEGs in mitochondrial dynamics and mitophagy based on transcriptome of GCs from WT and *Lrrc4^−/−^* mice. B) Representative fluorescent image showing co‐expression of mitochondria (red) and DRP1 (green) in NC, *LRRC4^−/−^* and Rescue KGN cells. Scale bar, 10 µm; magnified scale bar, 2 µm. C) The PCC and MCC analysis on the co‐expression of mitochondria and DRP1 signals in NC, *LRRC4^−/−^* and Rescue KGN cells (*n* = 30 biological replicates). D) Representative fluorescent image showing co‐expression of mitochondria (red) and MFN2 (green) in NC, *LRRC4^−/−^* and Rescue KGN cells. Scale bar, 10 µm; magnified scale bar, 2 µm. E) The PCC and MCC analysis on the co‐expression of mitochondria and MFN2 signals in NC, *LRRC4^−/−^* and Rescue KGN cells (*n* = 30 biological replicates). F) Representative fluorescent image showing co‐expression of mitochondria (red) and LC3B (green) in NC, *LRRC4^−/−^* and Rescue KGN cells. Scale bar, 10 µm; magnified scale bar, 2 µm. G) The PCC and MCC analysis on the co‐expression of mitochondria and LC3B signals in NC, *LRRC4^−/−^* and Rescue KGN cells (*n* = 30 biological replicates). H) Representative fluorescent image showing co‐expression of mitochondria (red) and LysoTracker (green) in NC, *LRRC4^−/−^* and Rescue KGN cells. Scale bar, 10 µm; magnified scale bar, 2 µm. I) The PCC and MCC analysis on the co‐expression of mitochondria and LysoTracker signals in NC, *LRRC4^−/−^* and Rescue KGN cells (*n* = 30 biological replicates). J) Representative fluorescent image showing co‐expression of mitochondria (red) and p62 (green) in NC, *LRRC4^−/−^* and Rescue KGN cells. Scale bar, 10 µm; magnified scale bar, 2 µm. K) The PCC and MCC analysis on the co‐expression of mitochondria and p62 signals in NC, *LRRC4^−/−^* and Rescue KGN cells (*n* = 30 biological replicates). L) Representative fluorescent images of mitophagic activity by mt‐Keima assays in NC, *LRRC4^−/−^* and Rescue KGN cells (*n* = 6 biological replicates). Cells treated with FCCP are positive controls. Scale bar, 10 µm. M) Representative electron microscopy images of mitochondria in NC, *LRRC4^−/−^* and Rescue KGN cells. Red arrow, normal mitochondria; yellow arrow, endoplasmic reticulum; green arrow, mitophagosome; blue arrow, autolysosome; black arrow, lysosome. Scale bar, 2 µm; magnified scale bar, 500 nm. N) Number of mitophagosome and autolysosome in NC, *LRRC4^−/−^* and Rescue KGN cells per microscopic field (*n* = 6 biological replicates). *^##^p* < 0.01 means NC versus *LRRC4^−/−^*. Results are calculated with one‐way ANOVA. Data are presented as mean ± SEM. **p < 0.01, ***p < 0.001, ****p < 0.0001.

Mitochondrial dynamics refers to the changing processes of fission, fusion, mitophagy, and transport, which affect the morphology, quality, quantity, distribution, and function of mitochondria within cells. Accordingly, we investigated whether the disruption of mitochondrial dynamics was involved in LRRC4‐induced aberrant mitochondrial remodeling. First, we determined the expression of key regulators of mitochondrial fission and fusion. The results revealed that LRRC4 knockout dramatically changed the expression of these key genes, especially *Dnm1l*, *Mfn2*, *Sqstm1*, and *Map1lc3a* (**Figure** **6**A). Immunofluorescence staining revealed that LRRC4 deficiency not only increased the fluorescence intensity of dynamin‐related protein 1 (DRP1, encoded by *DNM1L*) but also promoted DRP1 translocation from the cytosol to mitochondria (Figure 6B,C), whereas the expression of mitofusin 2 (MFN2) significantly decreased, with rare localization to mitochondria following LRRC4 knockout. Reintroduction of *LRRC4* restored the balance between mitochondrial fission and fusion (Figure 6D,E).

Mitophagy is a specialized form of autophagy that removes and degrades damaged mitochondria divided by mitochondrial fission to maintain mitochondrial quantity and quality. Mitochondrial fission and mitophagy work in concert to protect mitochondrial homeostasis. The activation of DRP1 has been found to promote mitophagy. Next, we investigated the role of LRRC4 in regulating mitophagy. As shown in Figure 6A, a heatmap of DEGs related to mitophagy revealed substantial differences between the groups. The coexpression of mitochondria with LC3B, p62, and LysoTracker was then observed by confocal microscopy. Immunofluorescence analyses revealed that LRRC4 knockout promoted the formation of mitophagosomes and mitolysosomes, as indicated by the colocalization of mitochondria with LC3 and LysoTracker (Figure 6F‒I). The decreased levels of p62 in the mitochondria and cytoplasm of *LRRC4^−/−^* KGN cells also indicated active mitophagy with unimpeded autophagic flux (Figure 6J,K). mt‐Keima assays were subsequently conducted to analyze the mitophagic activity quantitatively. The elevated red fluorescence intensity in LRRC4‐KO cells demonstrated increased formation of mitolysosomes and increased autophagic flux (Figure 6L). The reintroduction of LRRC4 restored overactivated mitophagy to normal conditions. TEM further confirmed the stimulatory effects of LRRC4 deficiency on mitophagy, as more mitophagosomes and autolysosomes were observed in *LRRC4^−/−^* cells than in control cells (Figure 6M,N).

Taken together, these findings indicate that LRRC4 is essential for maintaining mitochondrial homeostasis by balancing mitochondrial dynamics. LRRC4 deficiency results in excessive mitochondrial fission and mitophagy, leading to damaged mitochondrial structure and nonselective clearance.

### 2.6 LRRC4 Remodels Metabolism by Promoting YAP Degradation

**Figure 7:**
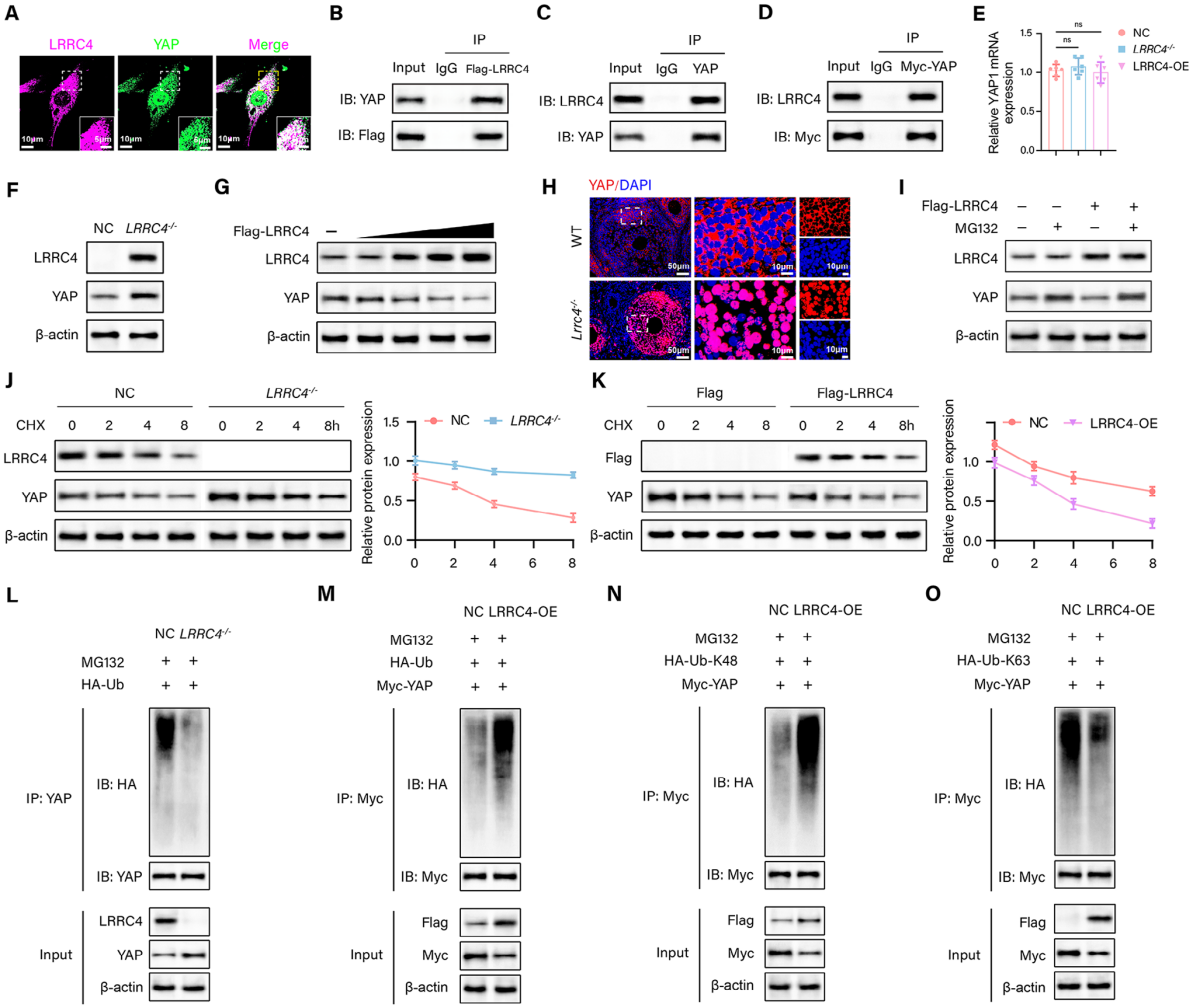
LRRC4 promotes YAP ubiquitination degradation. A) Representative fluorescent image showing co‐expression of LRRC4 (violet) and YAP (green) in KGN cells. Scale bar, 10 µm; magnified scale bar, 5 µm. B) Co‐IP assays showing the interaction of exogenous LRRC4 and YAP in KGN cells (*n* = 3 biological replicates). C and D) Co‐IP assays showing the interaction of exogenous LRRC4 with endogenous (C) and exogenous (D) YAP in KGN cells (3 independent experiments) in KGN cells (*n* = 3 biological replicates). E) Relative mRNA expression of YAP in NC, *LRRC4^−/−^* and LRRC4‐OE KGN cells (*n* = 3 biological replicates). LRRC4‐OE KGN cells refer to KGN cells transfected with FLAG‐LRRC4 expression vector. Results are calculated with one‐way ANOVA. F) Protein expression of YAP in NC and *LRRC4^−/−^* KGN cells (*n* = 3 biological replicates). G) Protein expression of YAP in KGN cells transfected with the increasing amount of FLAG‐LRRC4 expression vector (*n* = 3 biological replicates). H) Representative fluorescent images of YAP (red) in NC and *LRRC4^−/−^* KGN cells. Scale bar, 50 µm; magnified scale bar, 10 µm. I) Protein expression of YAP in LRRC4‐OE KGN cells treated with or without the proteasome inhibitor MG132 (20 µM, 6 h) (*n* = 3 biological replicates). J) Protein expression of YAP in NC and *LRRC4^−/−^* KGN cells treated with 30 µg mL^−1^ for the indicated times (*n* = 3 biological replicates). K) Protein expression of YAP in control and LRRC4‐OE KGN cells treated with 30 µg mL^−1^ CHX for the indicated times (*n* = 3 biological replicates). L) The ubiquitination of YAP in NC and *LRRC4^−/−^* KGN cells (*n* = 3 biological replicates). M) The ubiquitination of YAP in NC and LRRC4‐OE KGN cells (*n* = 3 biological replicates). N and O) K48‐linked (N) and K63‐linked (O) YAP polyubiquitination in NC and LRRC4‐OE KGN cells (*n* = 3 biological replicates). Data are presented as mean ± SEM. ns, not significant.

Emerging studies indicate that YAP1 regulates cell fate determination, mitochondrial function, energy metabolism, follicular activation, growth and differentiation, and steroidogenesis and plays a critical role in ovarian development. Hence, we hypothesized that LRRC4 maintains metabolic homeostasis by modulating YAP activity. We initially verified the coexpression of LRRC4 and YAP within the cytoplasm by immunofluorescence (**Figure** **7**A). Coimmunoprecipitation (co‐IP) assays further validated the direct interaction between LRRC4 and YAP (Figure 7B‒D). Moreover, LRRC4 knockout dramatically upregulated YAP protein expression (Figure 7F), whereas LRRC4 overexpression profoundly downregulated YAP expression in a dose‐dependent manner, which was reversed by the proteasome inhibitor MG132 (Figure 7G,H). However, relative *YAP1* mRNA expression remained unaffected (Figure 7E), indicating the post‐transcriptional regulation of YAP by LRRC4. In addition, the absence of LRRC4 promoted the nuclear translocation of YAP in GCs (Figure 7I). We subsequently explored the effects of LRRC4 on YAP stability by adding the protein synthesis inhibitor cycloheximide (CHX) and reported that LRRC4 knockout prolonged the half‐life of YAP (Figure 7J), whereas the stability of YAP decreased when LRRC4 was overexpressed (Figure 7K). These data strongly suggest that LRRC4 mediates YAP expression through the ubiquitin‐proteasome pathway. Next, we examined the effects of LRRC4 in regulating the ubiquitination level of YAP. As shown in Figure 7L,M, LRRC4 depletion significantly inhibited ubiquitinated YAP, while LRRC4 overexpression markedly increased the ubiquitination of YAP. Polyubiquitination, particularly at lysine residues 48 (K48) and 63 (K63), plays a pivotal role in the regulation of protein homeostasis and cellular signaling. Therefore, we further performed a ubiquitination assay with HA‐Ub‐K48 and HA‐Ub‐K63 to define the type of ubiquitin chain responsible for the LRRC4‐induced instability of YAP. LRRC4 effectively increased the K48‐linked ubiquitin chain but attenuated the K63‐linked ubiquitin chain on YAP (Figure 7N,O). These results demonstrate that LRRC4 promotes the degradation of YAP by increasing its K48‐linked ubiquitination and suppressing its K63‐linked ubiquitination, thereby inhibiting its transcriptional activity.

To further investigate whether LRRC4 targeted YAP to maintain the homeostasis of mitochondria and metabolism, we transfected YAP siRNA into *LRRC4^−/−^* KGN cells. Notably, YAP disruption suppressed the overactivation of mitochondrial fission and mitophagy induced by LRRC4 deficiency (Figure S5A, Supporting Information). Immunofluorescence demonstrated that si‐YAP significantly inhibited the coexpression of mitochondria with DRP1 and LC3 (Figure S5B,D; Supporting Information) but promoted the recruitment of MFN2 and p62 to mitochondria (Figure S5C,E; Supporting Information). Similarly, mt‐Keima assays further confirmed that mitophagy was inhibited in the absence of YAP (Figure S5F, Supporting Information). We then assessed the impact of YAP on metabolic disorders induced by LRRC4 deletion. Cells treated with si‐YAP presented normalized basal respiration, ATP production, and maximal oxygen consumption (Figure S5G‒J, Supporting Information), suggesting that disrupted YAP activity could reverse the impaired mitochondrial OXPHOS induced by LRRC4 knockout. With respect to glycolysis, si‐YAP addition resulted in a reduction in the expression of key genes related to glycolysis (Figure S5K, Supporting Information) and inhibited glycolytic activity compared with those in *LRRC4^−/−^* cells (Figure S5L‒N, Supporting Information). In addition, si‐YAP significantly upregulated *FSHR* and *CYP19A1* expression but inhibited *WT1* expression (Figure S6, Supporting Information). These data further confirmed that LRRC4 targeted YAP to maintain mitochondrial homeostasis and regulate metabolism, thereby rescuing the impaired GC differentiation.

### 2.7 Targeting LRRC4 Normalizes the Ovarian Development of POI Model Mice

**Figure 8:**
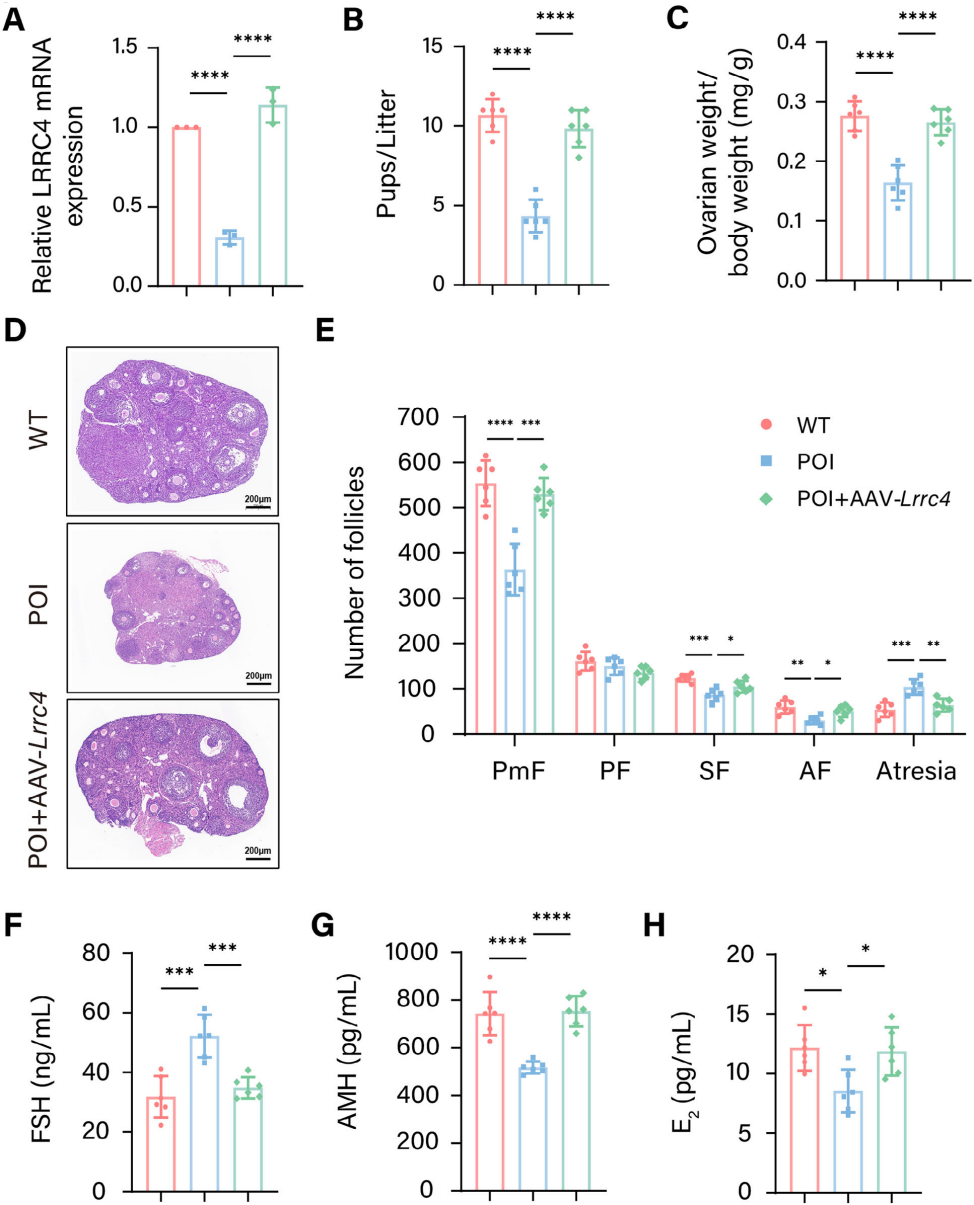
Targeting LRRC4 improves the ovarian development of POI mice. A) Relative mRNA expression of LRRC4 in ovaries from WT, POI, and POI+AAV‐*Lrrc4* mice (*n* = 3 biological replicates). B) Average litter size of WT, POI and POI+AAV‐ *Lrrc4* mice (*n* = 6 mice per group). C) Ovary index values of WT, POI, and POI+AAV‐ *Lrrc4* mice (*n* = 6 mice per group). D) Representative images of ovarian morphology from WT, POI, and POI+AAV‐ *Lrrc4* mice. Scale bar, 200 µm. E) Numbers of follicles in different developmental stages per ovary in WT, POI, and POI+AAV‐ *Lrrc4* mice (*n* = 6 mice per group). F, G) Serum levels of FSH (F), AMH (G), and E_2_ H) in WT, POI, and POI+AAV‐ *Lrrc4* mice (*n* = 6 mice per group). Results are calculated with one‐way ANOVA. Data are presented as mean ± SEM. *p < 0.05, **p < 0.01, ***p < 0.001, ****p < 0.0001.

To determine whether LRRC4 treatment can alleviate ovarian insufficiency, we established a POI‐like mouse model by CTX and reinforced LRRC4 via the intraperitoneal injection of adeno‐associated virus (AAV)–LRRC4. A substantial decrease in LRRC4 was observed in the ovaries of the POI‐like mice (**Figure** **8**A). Supplementation with LRRC4 significantly alleviated the ovarian insufficiency phenotype, as indicated by increased fertility (Figure 8B), normalized ovarian morphology (Figure 8C,D), increased antral follicle numbers, and decreased atretic follicle numbers (Figure 8E). Consistently, the levels of serum FSH, AMH, and E_2_ also returned to normal after LRRC4 treatment (Figure 8F‒H). Taken together, these data suggest that targeting LRRC4 could normalize ovarian development and alleviate key features of POI.

## 3 Discussion

As a key presentation of pathological ovarian aging, POI leads to accelerated systematic aging and severely compromises women's health and well‐being. POI is multifactorial and highly heterogeneous in etiology. A wide spectrum of causes has been recognized, including genetic, autoimmune, infectious, and iatrogenic causes. Nevertheless, the cause in the majority of cases remains unclear. Genetic factors have long been considered critical components of POI etiology and account for ≈20%–25% of patients.^[^ ^32^, ^33^, ^34^ ^]^ Over the past decades, many genes have emerged as POI candidates, but only a minority have been proven causative by functional validation. These include genes involved in primordial germ cell migration and proliferation (*NANOS3*), cell death (*PGRMC1*, *FMR1*), DNA damage repair (*MSH4/5*, *MCM8/9*), homologous recombination (*SPIDR*), meiosis (*STAG3*, *SYCE1*), oocyte‐specific transcription factors (FIGLA, NOBOX), other transcription factors affecting folliculogenesis (*NR5A1*, *WT1*, *FOXL2*), transforming growth factor‐β (TGF‐β) superfamily (*BMP15*, *GDF9*), hormone and receptors (*FSHR*, *AMH*, *AMHR2*) and mRNA transcription and translation (*eIF4ENIF1*, *KHDRBS*). None, however, could fully explain the disorder due to the remarkable heterogeneity in the etiology, as only a limited number of genes have been implicated in more than 5% of cases, highlighting the urgent need for novel insights into the pathogenesis.^[^ ^35^ ^]^ However, current studies predominantly emphasized oocyte‐centric mechanisms or transcriptional dysregulation, with granulosa cell‐autonomous regulation of mitochondrial homeostasis and metabolic adaptation throughout follicular development largely overlooked. Moreover, the interplay between mitochondrial dynamics and ovarian reserve maintenance remains unclear, limiting therapeutic strategies targeting follicular microenvironmental homeostasis. In this study, we identify LRRC4 as a novel regulator of folliculogenesis by triggering GC differentiation. This finding bridges the gap between mitochondrial dynamics and metabolic regulation in POI. Mechanistically, LRRC4 specifically coordinates mitochondrial fission and fusion in GCs by promoting the K48‐linked ubiquitination degradation of YAP, thereby preventing excessive mitophagy and maintaining metabolic homeostasis. Distinct from the roles of *BMP15* and *FOXL2* in the transcriptional networks, we focus on investigating the vital contribution of mitochondrial metabolic homeostasis to oocyte maturation within the follicular microenvironment. Importantly, targeting LRRC4 restores follicular development and ovarian function in POI model mice. Collectively, our study on LRRC4 not only expands the genetic landscape of POI but also enriches a mechanistic link between mitochondrial dysfunction and ovarian reserve depletion, providing a novel target for diagnosis and treatment.

Oogenesis and folliculogenesis are processes with high energy demands. Multiple studies have demonstrated that GCs can not only transfer ATP directly to oocytes through gap junctions but also provide essential metabolites for the energy metabolism of oocytes.^[^ ^36^, ^37^, ^38^ ^]^ With high glucose uptake and metabolic efficiency, GCs can produce a large amount of pyruvate and lactate from glucose metabolism as the main energy sources. Conversely, oocytes fail to utilize glycolysis because they lack multiple transporters and enzymes, and they rely mainly on energy substrates delivered from GCs for the TCA cycle.^[^ ^39^, ^40^ ^]^ Recent clinical trials have reported disturbed metabolism in POI patients;^[^ ^41^, ^42^, ^43^, ^44^, ^45^, ^46^, ^47^ ^]^ however, these studies have focused only on the plasma metabolome, and cell‐specific metabolic characteristics, especially the dynamic changes in energy metabolic patterns and substrate utilization in GCs in POI patients, are largely overlooked. In our study, metabolic reprogramming characterized by increased glycolysis and attenuated mitochondrial aerobic respiration was found both in GCs from LRRC4 deficiency‐induced POI model mice and in those from LRRC4‐KO KGN cells, which is consistent with the metabolic alterations in women with ovarian aging. Owing to mitochondrial dysfunction, increased glycolysis acts as a compensatory pathway for ATP production, whereas long‐term increased glycolysis leads to the accumulation of lactate, exacerbating mitochondrial damage. The PPP is a glucose oxidative degradation pathway that produces NADPH and ribose‐5‐phosphate. NADPH is involved in biosynthesis and maintains cytoplasmic integrity and redox status. Ribose 5‐phosphate can form phosphoribosyl pyrophosphate (PRPP), which is essential for DNA synthesis in oocytes.^[^ ^48^, ^49^ ^]^ Interestingly, we also detected reduced activity of the PPP in GCs lacking LRRC4, providing an explanation for the oxidative stress and defective oocyte maturation induced by LRRC4 deficiency.

Mitochondria are highly plastic and dynamic organelles that are essential for cellular metabolism, stress responses, and homeostasis maintenance.^[^ ^50^, ^51^ ^]^ Mitochondria undergo fission and fusion in response to various stressors and metabolic needs. These dynamic changes determine the morphology, distribution, quality, and quantity of mitochondria and are critical for maintaining mitochondrial structure and function.^[^ ^52^, ^53^ ^]^ A healthy mitochondrial network ensures follicular development and ovarian function. Many studies have shown that POI is strongly associated with mitochondrial dysfunction,^[^ ^54^, ^55^, ^56^, ^57^ ^]^ but the specific mitochondrial mechanisms involved are not fully understood. In this study, impaired mitochondrial function was confirmed in both LRRC4‐KO GCs and KGN cells, characterized by a defective mitochondrial respiratory chain, thus exacerbating mtROS accumulation and MMP collapse, leading to reduced OXPHOS activity. The structural integrity of mitochondria, especially their crista structure and density, influences the capacity of the mitochondrial respiratory chain.^[^ ^58^, ^59^ ^]^ Assessments of mitochondrial morphology revealed that the loss of LRRC4 resulted in swollen and fragmented mitochondria with broken and decreased cristae, indicating a damaged mitochondrial ultrastructure and disrupted mitochondrial dynamics with increased mitochondrial fission, which was further verified by the increased expression of DRP1 in mitochondria. Regulators of mitochondrial fission, such as DRP1, are crucial for the initiation of mitophagy.^[^ ^60^, ^61^, ^62^ ^]^ Our results revealed that there were more mitophagosomes and autolysosomes in the LRRC4‐KO cells than in the control cells, together with increased autophagic flux, which suggested that mitophagy was excessively activated when LRRC4 was absent. Here, we found that LRRC4 is essential for maintaining mitochondrial homeostasis by balancing mitochondrial dynamics and that LRRC4 deficiency triggers excessive mitochondrial fission and mitophagy, leading to mitochondrial damage.

In contrast to the dynamic expression pattern of YAP reported by previous studies, we detected clear nuclear YAP in LRRC4‐KO GCs, which exhibited a differentiation block phenotype. Notably, our results demonstrated that LRRC4 changed the protein expression of YAP rather than its transcriptional level, indicating that the effects of LRRC4 on YAP are post‐translational. YAP was shown to undergo degradation through K48‐linked ubiquitination, while K63‐linked ubiquitination of YAP mediates nondegradative processes and promotes the transcriptional activity of YAP.^[^ ^63^, ^64^ ^]^ Here, we found that LRRC4 increased K48‐linked YAP polyubiquitination and decreased K63‐linked YAP polyubiquitination, which is consistent with the findings of decreased YAP protein levels and attenuated YAP transcriptional activity. The inhibition of YAP activity in KGN cells further confirmed that YAP is a crucial mediator of the LRRC4 deficiency‐induced disruption of follicular development and mitochondrial homeostasis. In addition, YAP can interact with other signaling pathways, such as the Hippo, Wnt, and TGF‐β signaling pathways, to maintain follicular homeostasis. The Hippo pathway has been implicated in governing folliculogenesis and steroidogenesis by regulating the transcriptional activity of YAP through phosphorylation‐mediated nuclear localization.^[^ ^18^ ^]^ Emerging evidence indicates that there is a negative feedback regulation between LATS2 and YAP, where overactivated YAP upregulates LATS2, and further induces ovarian cell senescence.^[^ ^65^ ^]^ The Wnt pathway is another critical regulator of ovarian function and YAP has been reported to synergize with β‐catenin (core effector of the Wnt pathway) to enhance target gene expression.^[^ ^66^ ^]^ The TGF‐β pathway plays a pivotal role in folliculogenesis by regulating granulosa cell proliferation, apoptosis, and steroidogenesis. Notably, TGF‐β can modulate YAP activity through Smad‐dependent mechanisms.^[^ ^67^ ^]^ Our findings that LRRC4 promotes YAP degradation suggest potential cross‐talk between LRRC4/YAP and these signaling pathways, where LRRC4 could indirectly affect their activity by regulating YAP stability. These complex molecular interactions highlight the pivotal role of LRRC4/YAP as a central regulatory hub that orchestrates multiple signaling pathways to modulate folliculogenesis. Future research should focus on delineating the specific molecular mechanisms underlying these interactions, with particular emphasis on elucidating how LRRC4/YAP mediates the complex processes regarding follicular development and ovarian function maintenance, thereby identifying novel targets for the diagnosis and management of POI.

Our study has several limitations. First, although preliminary evidence from published human single‐cell and bulk transcriptomic datasets suggests a positive correlation between LRRC4 expression and ovarian reserve, all findings are currently confined to preclinical models (e.g., mice and granulosa cell lines) with the absence of direct validation in clinical samples, which limits the clinical relevance of our conclusions. In addition, the lack of standardized, high‐sensitivity techniques for quantitatively assessing LRRC4 in vivo (e.g., in serum or tissue) represents a critical translational gap. Future studies should conduct clinical trials and develop related test kits to confirm the role of LRRC4 in POI patients, meanwhile defining the clinical diagnostic thresholds of LRRC4 to predict ovarian reserve and menopausal age, which will contribute to the establishment of early warning for ovarian aging, as well as early diagnosis and timely treatment for diseases related to ovarian insufficiency. Second, post‐translational modifications (PTMs) include SUMOylation, methylation, acetylation, O‐GlcNAcylation, and lactylation. Future investigations are needed to explore whether LRRC4 regulates other PTMs of YAP, particularly lactylation, as LRRC4 deficiency leads to the accumulation of lactate. Third, our findings indicate that LRRC4 deficiency is associated with a significant reduction in MPC2, which is involved in transporting pyruvate into the mitochondria. Further study is needed to elucidate the role of LRRC4 in the transportation of energy substrates.

In summary, our study reveals a novel mechanism of POI control by LRRC4, thus providing a promising target for the prevention and treatment of POI. Our results map the metabolic landscape of GCs in POI and reveal the critical role of LRRC4 in maintaining metabolic homeostasis by balancing mitochondrial fission and fusion, revealing a new regulator of GC metabolism.

## 4 Experimental Section

### Mice

*Lrrc4^−/−^* mice were generated via CRISPR/Cas‐mediated genome engineering by Cyagen Biosciences, Inc. One exon was identified, with the ATG start codon in exon 1 and the TGA stop codon in exon 1 (Transcript Lrrc4‐201: ENSMUST00000062304). Exon 1 was selected as the target site. The region contains a 1959 bp coding sequence. The resulting mouse was sequenced, which confirmed a 5.5 kb deletion. For POI model mice, female C57BL/6 mice were intraperitoneally injected with 100 mg k^−1^g CTX for the first day and then with 10 mg k^−1^g d CTX for 13 days. The mice in the control group were injected with an equal volume of saline. AAV‐*Lrrc4* (1.5 × 10^10^ genome copy particles) was injected into the ovaries of mice with POI to increase the expression of LRRC4. The mice were housed in groups with ad libitum access to food and water under a 12‐hour light/12‐hour dark cycle in a temperature‐ and humidity‐controlled environment (20–22 °C, 45%–55%). All comparisons were made between littermates. All animal procedures were conducted according to the National Institutes of Health Guide for the Care and Use of Laboratory Animals (NIH Publication No. 85‐23; revised 1985) and following local ethical approval at Central South University (Ethical number: 2023‐KT050).

### Cell Lines

The KGN cell line, a human ovarian granulosa‐like tumor cell line that maintains the physiological characteristics of GCs, was purchased from RIKEN Bioresource Centre (RCB1154, Tsukuba, Japan). LRRC4 knockout (*LRRC4^−/−^*) cell lines were generated using the CRISPR/Cas9 system. For the LRRC4 rescue cells, *LRRC4^−/−^* KGN cells were transduced with a lentiviral vector carrying the coding sequence for LRRC4. Single clone cells were selected with puromycin and subjected to Western blotting. The cells were cultured in Dulbecco's modified Eagle's medium (DMEM)/F12 supplemented with 10% fetal bovine serum, 100 U mL^−1^ penicillin, and 100 µg mL^−1^ streptomycin in 5% CO_2_ at 37 °C.

### Fertility Test

Female mice were continuously mated with fertile wild‐type male mice from the ages of 8 to 36 weeks (female: male = 1:1, *n* = 5 per group). The numbers of pups and litters were recorded.

### Follicle Quantification

Follicles were scored in every fifth section, and in each section, only those follicles with a clear oocyte nucleus were counted and multiplied by five to calculate the total number of follicles in an ovary. Ovarian follicles at different developmental stages were identified according to the well‐accepted standards established by Peterson and Peters.

### Oocyte and GC Collection

Three‐week‐old female mice were intraperitoneally injected with 10 IU PMSG followed by 10 IU hCG 48 h later. Oocytes were collected from oviductal ampullae at ≈13–14 h after hCG injection and briefly exposed to 1 mg mL^−1^ hyaluronidase to remove GCs.

Three‐week‐old female mice were sacrificed after being intraperitoneally injected with 10 IU PMSG for 48 h. The ovaries were separated from the ovarian capsule in prechilled phosphate‐buffered saline (PBS) (10 mmol L^−1^, pH 7.4) under a stereomicroscope. The GCs were released from the follicles by needle puncture. After the oocytes were removed, the remaining liquid was centrifuged to obtain GCs.

### Histological Assessment and Immunofluorescence

Ovaries were fixed in 4% paraformaldehyde, dehydrated, embedded in paraffin, and then sectioned at a thickness of 5 µm for hematoxylin and eosin staining or Masson staining.

For immunofluorescence staining, the embedded sections were dewaxed, treated with citrate for antigen retrieval, and incubated with the indicated primary antibodies at 4 °C overnight. The cells were immersed in 4% paraformaldehyde and blocked with 10% goat serum, followed by incubation overnight with the primary antibody at 4 °C. The samples were subsequently incubated with the corresponding secondary antibodies. The nuclei were stained with DAPI. After being sealed with an antiquenching mounting medium, the samples were observed by a confocal microscope (LSM 980+Airyscan 2, Carl Zeiss). The following antibodies and dyes were used: anti‐LRRC4 antibody (Bioss, bs‐1974R), anti‐FOXL2 antibody (Abcam, ab246511), anti‐DDX4 antibody (Abcam, ab13840), anti‐γH2AX antibody (Cell Signaling Technology, #9718), anti‐FOXO3A (Cell Signaling Technology, #2497), anti‐p‐RPS6 antibody (Cell Signaling Technology, #4858), anti‐caspase 3 antibody (Cell Signaling Technology, #9661), anti‐PCNA antibody (Cell Signaling Technology, #13 110), anti‐FSHR antibody (Abcam, ab113421), anti‐WT1 antibody (Abcam, ab89901), anti‐DRP1 antibody (Abcam, ab184247), anti‐MFN2 antibody (Cell Signaling Technology, #9482), anti‐MFN2 antibody (Cell Signaling Technology, #9482), anti‐LC3B antibody (Abcam, ab192890), anti‐p62 antibody (Abcam, ab109012), anti‐YAP antibody (Abcam, ab52771), MitoTracker Deep Red (Thermo Fisher Scientific, M22426), MitoSOX (Thermo Fisher Scientific, M36008), MitoTracker Red CMXRos (Thermo Fisher Scientific, M7512), Alexa Fluor 488 (Invitrogen, A11029, A11034), Alexa Fluor 568 (Invitrogen, A11031, A11036) and Alexa Fluor 647 (Invitrogen, A21236, A21245).

Colocalization was measured with PCC and MCC using Coloc2 in Fiji. The PCC values ranged from one for a positive correlation between two images to −1 for a negative correlation between two images. Among the MCC coefficients, the values ranged from 0 for no colocalization to 1 for complete colocalization.

### Enzyme‐Linked Immunosorbent Assay (ELISA)

After anesthetization with isoflurane, the eyeballs were removed to collect blood samples. The serum was divided after centrifugation for hormone detection according to the manufacturer's instructions. The absorbance (OD value) was measured by an enzyme‐labeled instrument at a wavelength of 450 nm to further calculate the sample concentration. The following ELISA kits were used: an FSH ELISA kit (Cloud‐clone, CEA830Mu), an AMH ELISA kit (Cloud‐clone, CEA228Mu), and an E_2_ ELISA kit (Cloud‐clone, CEA461Ge).

### RNA‐seq Analysis

RNA‐seq was performed on GCs isolated from 8‐week‐old *Lrrc4^−/−^* mice and littermate WT mice (*n* = 3 per genotype). Total RNA was isolated, and RNA‐seq was performed via an Illumina NextSeq 500, with the raw reads aligned to the mouse reference genome (Version mm10) using HISAT2. The read counts were normalized to feature counts to obtain the values of reads per kilobase per million (RPKM). Then, the z scores, which represent how many standard deviations one particular count was from the average count for that gene, and adjusted p values using Benjamini‒Hochberg correction were obtained. Here, an adjusted *p* value < 0.05 and log2 (fold change)>1 were considered as significantly changed. KEGG pathway analysis and GO enrichment analysis were performed using DAVID (https://david.ncifcrf.gov/summary.jsp). GSEA was performed (https://www.gsea‐msigdb.org/gsea/index.jsp).

### Real‐Time qPCR

Total RNA was extracted using TRIzol reagent (catalog T9108, TaKaRa) according to the manufacturer's protocol. The RNA concentrations were determined by a Nanodrop analyzer (Thermo Scientific). Complementary DNA samples were synthesized with a reverse‐transcription kit (Vazyme, R233). Real‐time PCR was performed using SYBR Green gene expression assays (Vazyme, R711). The experiments were performed with three independent biological replicates unless otherwise noted. For quantification analysis, gene expression was determined relative to that of GAPDH and normalized to that of the control groups. The primers were queried in PRIMERBANK (https://pga.mgh.harvard.edu/primerbank/), and the sequences were validated in the NCBI nucleotide database (https://blast.ncbi.nlm.nih.gov/Blast.cgi). The sequences of primers used are listed in Table S1 (Supporting Information).

### Metabolomic Analysis

The culture medium was gently aspirated, and the cells were collected in a glass centrifuge tube. After 20 µL of d4‐succinate internal standard solution and 1 mL of cooled methanol: water (80:20, v/v) solution were added, the cells were extracted by ultrasonication and vortexed for 1 min. The centrifuge tubes were incubated at −80 °C for 12 h to aid in cell division and protein precipitation, exposed to ultrasonication, and vortexed again for 1 min. After centrifugation at 12000 × g for 5 min at 4 °C, the supernatant was transferred to another glass centrifuge tube, dried under a gentle nitrogen stream, and then kept at −80 °C. The dried extracts were resuspended in 120 µL of acetonitrile:water (90:10, v/v) mixture. After centrifugation at 12000 × g for 5 min at 4 °C, a 100 µL volume was transferred to autosampler vials and analyzed for metabolomics and isotope tracing metabolic flux analysis via an LC‐MS system. The experiments used a Waters ACQUITY UPLC I‐Class plus/Thermo QE HF. Metabolites were separated on an ACQUITY UPLC HSS T3 column (100 × 2.1 mm, 1.8 µm particle size). Sample analysis was performed in negative ionization mode. Data analysis was conducted via functional metabolomics (FunMeta).

### Seahorse Extracellular Flux Assay

Glycolytic and mitochondrial stress tests were performed with a Seahorse XF96 Extracellular Flux Analyzer (Agilent Technologies) to measure the ECAR and the OCR. KGN cells were plated in an XFe24 plate at a density of ≈1 × 104 cells/well. The cell culture medium was removed and replaced with Seahorse XF assay medium, which contained 2 mmol L^−1^ glutamine, 10 mmol L^−1^ glucose, and 2 mmol L^−1^ sodium pyruvate. On the day of measurement, the cells were washed with XFe96 media and incubated in a CO_2_‐free incubator at 37 °C for 2 h to establish equilibration prior to loading. The following compounds were injected at a final concentration per well to determine the glycolysis and glycolytic capacity of the indicated KGN cells: 10 mM glucose (Sigma, G5767), 1 mM oligomycin (Selleck, S1478), and 50 mM 2‐DG (Selleck, S4701). Mitochondrial OXPHOS was evaluated as follows: 1 mM oligomycin (Selleck, S1478), 1 mM FCCP (Selleck, S8276), and 0.5 mM rotenone/antimycin A mixture. The reagents, test kits, and plates were purchased from Agilent Technologies.

### TEM

Cells were washed twice with PBS and trypsinized, and 10 million cells of each genotype were collected and spun down at low speed to form a firm pellet. High‐pressure freezing was performed via a Leica ICE high‐pressure freezer apparatus with a gold‐plated sample carrier (carrier A, 3 mm diameter, 100 µm deep; carrier B, flat side down). The sample carriers were lightly coated with 0.1% soy lecithin in chloroform to ensure a smooth and easy opening of the carrier without damaging the pellet. Carrier A was filled with well‐pelleted cells, and carrier B was placed on top with the flat side down before freezing at a programmed pressure of 2100 bars. After freezing, the sample pod was released automatically into a liquid nitrogen bath; the sample carrier was then separated from the sample pod using precooled fine‐tipped tweezers under liquid nitrogen and transferred to a Leica freeze‐substitution AFS2 set up in a 2 mL solution of cold dry absolute acetone (v/v) containing 1% osmium tetroxide. The AFS unit was slowly warmed from −90 to 0 °C (2 °C h^−1^), with the temperature being held at both −90 °C for a period of 15 h and thereafter at −60 and −30 °C for a period of 8 hours each. The samples were cleared of osmium by rinsing with absolute acetone (3 times × 5 min) and thereafter infiltrated with low‐viscosity resin at increasing concentrations of 30% and 66% for 4 h each and 100% overnight. Individual samples were embedded in 1 mL of 100% low‐viscosity resin and polymerized for 30 h at 60 °C. The resin blocks were tapered into a pyramid shape, polished using a target sample preparation unit (Leica TXP), and sectioned via a Leica ultramicrotome (UC7) with a diamond knife at low speed to obtain 60–75‐nm‐thick sections. Ultrathin sections were mounted on 200 mesh copper grids for imaging.

### Western Blot

Cells were lysed in RIPA buffer (catalog 89900, Thermo Fisher Scientific) supplemented with protease and phosphatase inhibitor cocktail (catalog 78440,Thermo Fisher Scientific). Protein concentrations were measured via a BCA protein assay kit (catalog 23225, Thermo Fisher Scientific). Equal concentrations of proteins were diluted in 1×SDS‒PAGE loading buffer and denatured at 95 °C for 10 min. Protein lysates were transferred onto an SDS polyacrylamide gel, subjected to electrophoresis, and transferred to a PVDF membrane. After being blocked in 5% milk for 1 h at room temperature, the membrane was incubated overnight with primary antibodies at 4 °C. The membrane was incubated with peroxidase‐conjugated secondary antibody for 1 hour at room temperature and then washed three times with PBST. The images were captured with a chemiluminescence detection system (Bio‐Rad). The experiments were performed with three independent biological replicates unless otherwise noted.

### Co‐IP

Cells were harvested and lysed in an immunoprecipitation lysis buffer. The total cell lysate was precleared with rabbit IgG for 2 h and subsequently immunoprecipitated with the indicated antibody at 4 °C overnight. Protein A/G PLUS‐Agarose beads (Santa Cruz) were then added to the lysates and incubated at 4 °C for 2 h. The immunocomplexes were washed with lysis buffer three times and separated by SDS‒PAGE. Immunoblotting was performed following standard procedures.

### Statistics and Reproducibility

All data were evaluated for normality using Shapiro‐Wilk tests (α = 0.10) prior to parametric analyses. Variance homogeneity was verified through Levene's test (α = 0.05). To address the potential inflation of Type I errors arising from multiple hypothesis testing, the Bonferroni method was applied for outcome analyses involving correlated variables. Normally distributed data were compared with two‐tailed Student's t‐tests or one‐way ANOVA. Two‐tailed Mann–Whitney U test and two‐tailed Kruskal–Wallis test were used when the data were not normally distributed. The results were considered statistically significant when *p *< 0.05. Pearson correlation analysis was used to determine the correlation between gene expression levels. The evaluated parameters in the groups are shown as the means and standard deviations of the means (SEMs) for each group. Each experiment was repeated at least three times unless otherwise specified. Statistical analyses were performed using GraphPad Prism 9.0 software.

## Conflict of Interest

The authors declare no conflict of interest.

## Supporting information



Supporting Information

## Data Availability

The data that support the findings of this study are available from the corresponding author upon reasonable request.
