# Supplementary material for: LRRC4 Deficiency Drives Premature Ovarian Insufficiency by Disrupting Metabolic Homeostasis in Granulosa Cells
Source: Adv Sci (Weinh). 2025 May 2;12(23):2417717. doi: 10.1002/advs.202417717 (PMC12199380; doi:10.1002/advs.202417717)
Supplement: Supplementary file 1 — Supporting Information [file ADVS-12-2417717-s001.docx]

Supporting Information

LRRC4 deficiency drives premature ovarian insufficiency by disrupting metabolic homeostasis in granulosa cells

Yujie Shang, Yunjun Li, Di Han, Kun Deng, Wei Gao, Minghua Wu*

**Table S1.** Primer sequences in this study

**Figure S1.** Ovarian morphology of 3-week-old mice

**Figure S2.** Follicular quantification of 16-month-old mice

**Figure S3.** The percentage of PB1 extrusion after 16 hours in vitro culture

**Figure S4.** LRRC4 deficiency provokes apoptosis and DNA damage in granulosa cells

**Figure S5.** LRRC4 targets YAP to remodel metabolism

**Figure S6.** LRRC4 targets YAP to promote the differentiation of granulosa cells

**Table S1** Primer sequences in the study

| **Gene** | **species identity** | **Forward Primer** | **Reveres Primer** |
| --- | --- | --- | --- |
| *GAPDH* | Human | GGTTGTCTCCTGCGACTTCA | TGGTCCAGGGTTTCTTACTCC |
| *FOXK1* | Human | CCCGCCTCCATCGTAACCTC | CGAGTTGGCAGATGTGGTGAC |
| *BPGM* | Human | GAATGGGTGCCTGTGGAAAGC | CCAGAGCCTCACTTGTTCTTCAC |
| *PHGDH* | Human | GTGCGGCTGCTGTCCTACC | GCTTCAGTCACATGCTGCTTCC |
| *LDHA* | Human | GATTCAGCCCGATTCCGTTACC | CATTCCACTCCATACAGGCACAC |
| *HK2* | Human | GGATGTGGTTGCTGTGGTGAAC | GCTGCCCGTGCCAACAATG |
| *PGAM1* | Human | GCATCTGGAGGGTCTCTCTGAAG | AAACTGCATGGGCTTGATAGGC |
| *PFKFB3* | Human | CGACAAATGCGACAGGGACTTG | TACACGATGCGGCTCTGGATG |
| *ACO2* | Human | CACGGATGGCAAGAAGTTCAGG | CTTGGGTGGGTGCTGGTAGG |
| *ATP5D* | Human | CGGCTCCTTCGGCATCCTG | CCGCTGCTCACGAAGTATTTGG |
| *COX6A1* | Human | AGCTCGCATGTGGAAGACTCTC | CTCTCGTGCTCTCCGTGGTG |
| *MDH1* | Human | GTGCCCTTCCCCTCCTGAAAG | TCTCTCCATGCCTTCCCTTCTTG |
| *NDUFA11* | Human | AGCCTACAGCACCACCAGTATTG | TGTCCAACCTTAGCCACTCCTTC |
| *OGDH* | Human | ACTGTGGCGACACTGAAGGG | GGTTATGTTGGTGAGCGGAACTC |
| *SDHB* | Human | GCAGTCCATAGAAGAGCGTGAG | TGTCTCCGTTCCACCAGTAGC |
| *UQCR10* | Human | CATCGTGGGCGTCATGTTCTTC | CAGGCGGGTCCAAGGTCAG |
| *SQSTM1* | Human | CGGCTGATTGAGTCCCTCTCC | CCGCTCCGATGTCATAGTTCTTG |
| *LAMP1* | Human | GGCTCTGTGGAGGAGTGTCTG | CCGACGAGGTAGGCGATGAG |
| *MAP1LC3A* | Human | GCCTTCTTCCTGCTGGTGAAC | GGGAGGCGTAGACCATATAGAGG |
| *MFN2* | Human | AGCAGATTACGGAGGAAGTGGAG | GGACTACTGGAGAAGGGTGGAAG |
| *DNM1L* | Human | CACAGGCAACTGGAGAGGAATG | TTGTGGACTGGCTGGCATAATTG |
| *LRRC4* | Human | TGCCGCCATGTTGATTGTCTTC | GATGTTGCTGCTGGGATGTCTTC |
| *YAP* | Human | GAACCGTTTCCCAGACTACCTTG | TTGGCATCAGCTCCTCTCCTTC |
| *MPC2* | Human | CCGAGAAATTGAGGCCGTTGTAC | CCATATCAGCCAATCCAGCACAC |
| *Gdf9* | Mouse | GGCCCCGCACAGGTACAACC | GCCGTACCGATGCCTGACCG |
| *Bmp15* | Mouse | ACCGCCCTCCTTGCTGACGA | TGCGGGTCAGCCGAACGATG |
| *Kitl* | Mouse | GAATCTCCGAAGAGGCCAGAA | GCTGCAACAGGGGGTAACAT |
| *Ngf* | Mouse | CCAGTGAAATTAGGCTCCCTG | CCTTGGCAAAACCTTTATTGGG |
| *Gdnf* | Mouse | GAGGCATCTGGTCACAGCGATAAG | ATGGCAGGCACTTGGAGTCTTAAC |
| *Sirt1* | Mouse | TGTTTCCTGTGGGATACCTGA | TGAAGAATGGTCTTGGGTCTTT |
| *Lrrc4* | Mouse | AGTGGCAAAGTGGAGATTGTTG | TGTTAGTGGGGTCTCGCTCC |

**
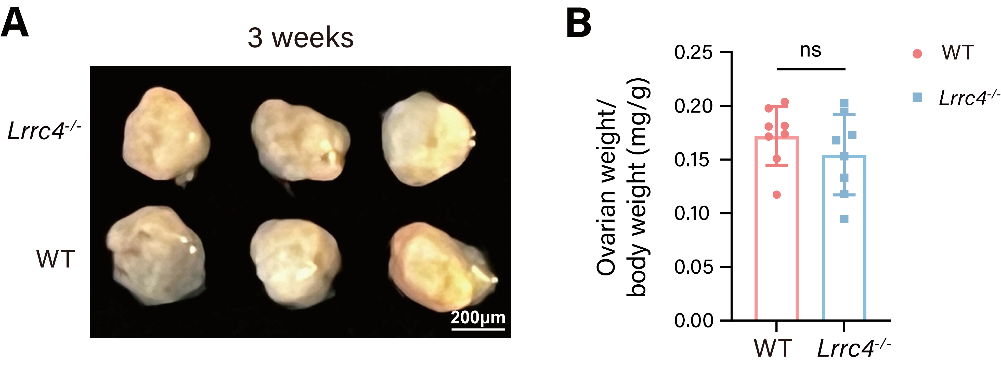
Figure S1.** Ovarian morphology of 3-week-old mice. (A) Representative images of ovarian morphology from 3-week-old WT and *Lrrc4^-/-^*. (B) Ovary index values of 3-week-old WT and *Lrrc4^-/-^* mice (n=8 mice per group).

**
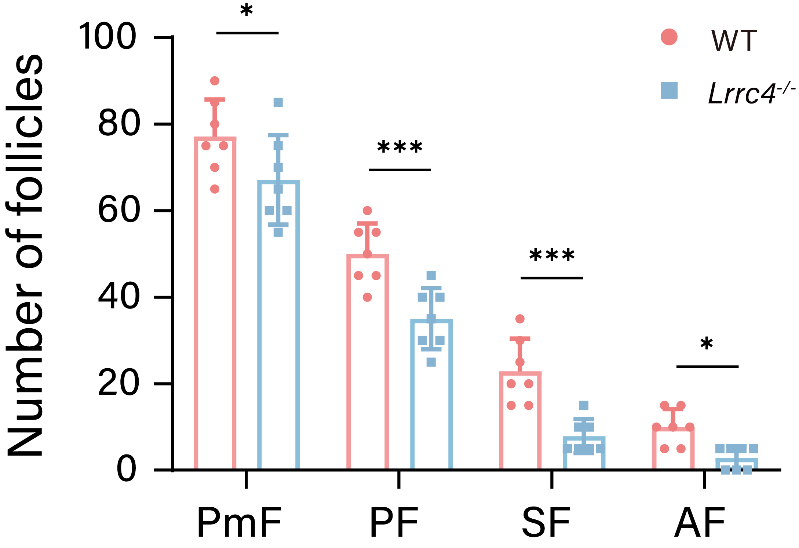
Figure S2.** Follicular quantification of 16-month-old mice. Numbers of follicles in different developmental stages per ovary from 16-month-old WT and *Lrrc4^-/-^* mice (n=7 mice per group). **P* < 0.05, ****P* < 0.001.

**
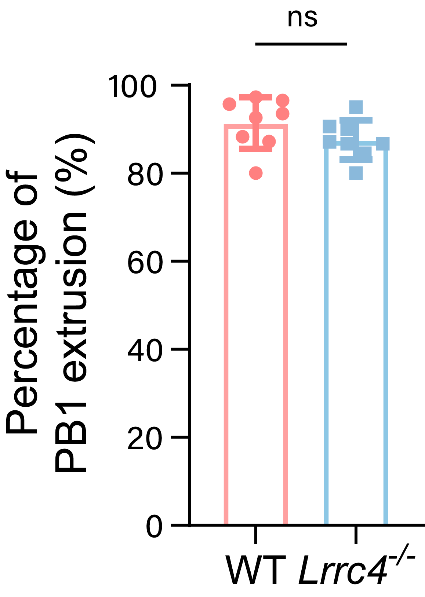
Figure S3.** The percentage of PB1 extrusion after 16 hours in vitro culture (n=8 mice per group). ns, not significant.

**
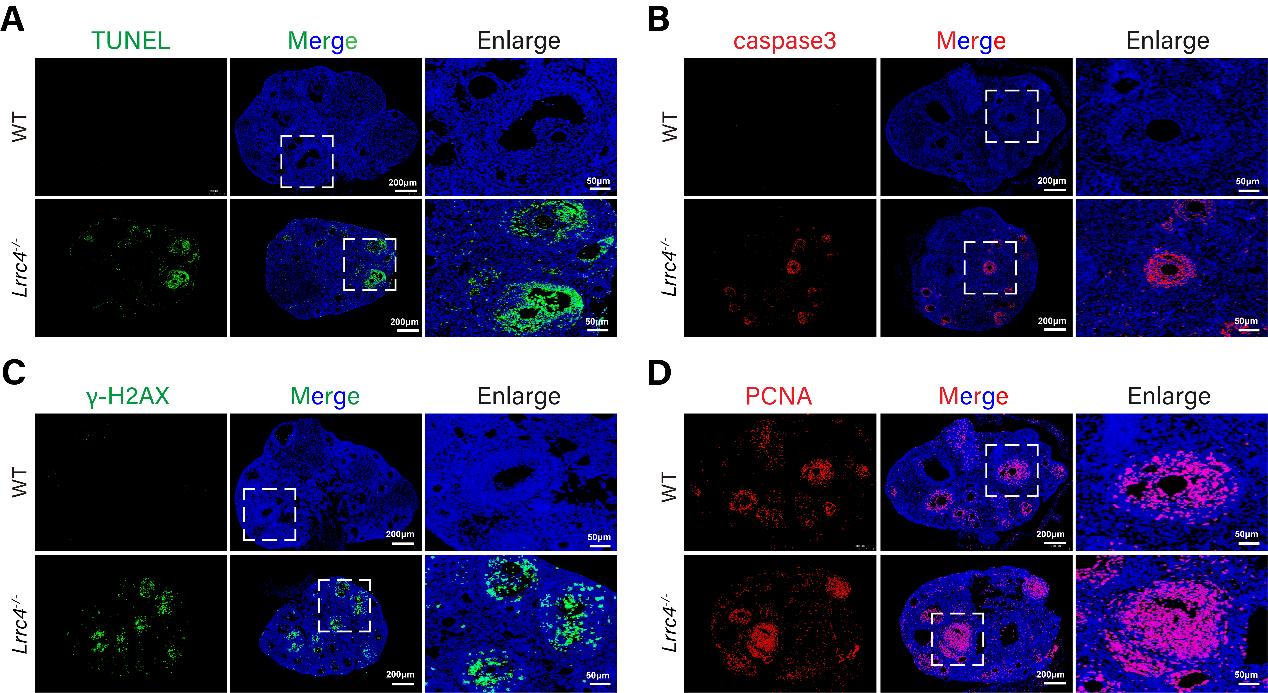
Figure S4.** LRRC4 deficiency provokes apoptosis and DNA damage in GCs. Representative fluorescent images of TUNEL (A), Caspase 3 (B), γ-H2AX (C) and PCNA (D) expression in GCs from 8-week-old WT and *Lrrc4^-/-^* mice. Scale bar, 200 μm; magnified scale bar, 50 μm. GCs, granulosa cells.

**
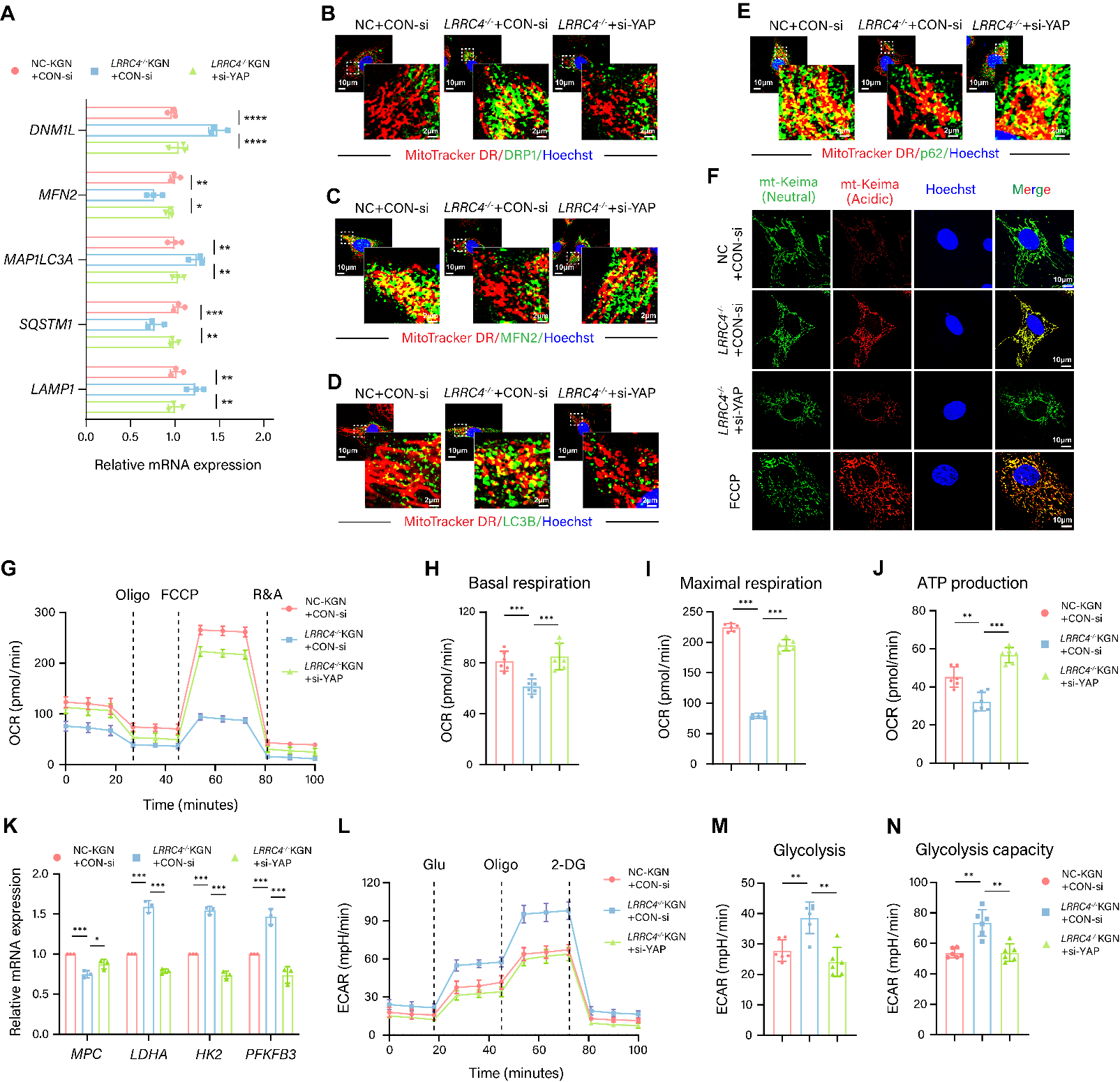
Figure S5.** LRRC4 targets YAP to remodel metabolism. (A) Relative mRNA expression of genes in mitochondrial dynamics and mitophagy in NC, *LRRC4^-/-^* and *LRRC4^-/-^*+si-YAP KGN cells (n=3 biological replicates). *LRRC4^-/-^*+si-YAP KGN cells refer to *LRRC4^-/-^* KGN cells transfected with si-YAP. (B) Representative fluorescent image showing co-expression of mitochondria (red) and DRP1 (green) in NC, *LRRC4^-/-^* and *LRRC4^-/-^*+si-YAP KGN cells. Scale bar, 10 μm; magnified scale bar, 2 μm. (C) Representative fluorescent image showing co-expression of mitochondria (red) and MFN2 (green) in NC, *LRRC4^-/-^* and *LRRC4^-/-^*+si-YAP KGN cells. Scale bar, 10 μm; magnified scale bar, 2 μm. (D) Representative fluorescent image showing co-expression of mitochondria (red) and LC3B (green) in NC, *LRRC4^-/-^* and *LRRC4^-/-^*+si-YAP KGN cells. Scale bar, 10 μm; magnified scale bar, 2 μm. (E) Representative fluorescent image showing co-expression of mitochondria (red) and p62 (green) in NC, *LRRC4^-/-^* and *LRRC4^-/-^*+si-YAP KGN cells. Scale bar, 10 μm; magnified scale bar, 2 μm. (F) Representative fluorescent images of mitophagic activity by mt-Keima assays in NC, *LRRC4^-/-^* and *LRRC4^-/-^*+si-YAP KGN cells. Cells treated with FCCP are positive controls. Scale bar, 10 μm. (G to J) Real-time seahorse OCR measurements for OXPHOS speed evaluation in NC, *LRRC4^-/-^* and *LRRC4^-/-^*+si-YAP KGN cells. (K) Relative mRNA expression of genes in glycolysis in NC, *LRRC4^-/-^* and *LRRC4^-/-^*+si-YAP KGN cells (n=3 biological replicates). (L to N) Real-time seahorse ECAR measurements for glycolysis in NC, *LRRC4^-/-^* and *LRRC4^-/-^*+si-YAP KGN cells. Data are presented as mean ± SEM. **P* < 0.05, ***P* < 0.01, ****P* < 0.001, *****P* < 0.0001.

**
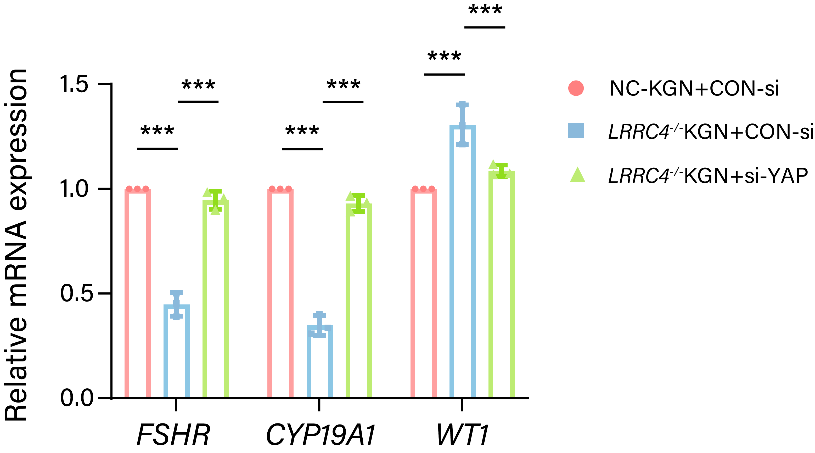
Figure S6.** LRRC4 targets YAP to promote the differentiation of granulosa cells. ****P* < 0.001.
